# OTUB1 promotes osteoblastic bone formation through stabilizing FGFR2

**DOI:** 10.1038/s41392-023-01354-2

**Published:** 2023-04-07

**Authors:** Qiong Zhu, Yesheng Fu, Chun-Ping Cui, Yi Ding, Zhikang Deng, Chao Ning, Fan Hu, Chen Qiu, Biyue Yu, Xuemei Zhou, Guan Yang, Jiang Peng, Weiguo Zou, Cui Hua Liu, Lingqiang Zhang

**Affiliations:** 1 State Key Laboratory of Proteomics, National Center for Protein Sciences (Beijing), Beijing Institute of Lifeomics, Beijing, 100850 China; 2 Lab of Orthopedics of Department of Orthopedics, Beijing Key Lab of Regenerative Medicine in Orthopedics, Chinese PLA General Hospital, Beijing, 100853 China; 3 Department of Endocrinology, The Second Medical Center & National Clinical Research Center for Geriatric Diseases, Chinese PLA General Hospital, Beijing, 100853 China; 4 School of Life Sciences, Hebei University, Baoding, Hebei, 071002 China; 5 State Key Laboratory of Cell Biology, Shanghai Institute of Biochemistry and Cell Biology, Center for Excellence in Molecular Cell Science, Chinese Academy of Sciences, University of Chinese Academy of Sciences, Shanghai, 200031 China; 6 CAS Key Laboratory of Pathogenic Microbiology and Immunology, Institute of Microbiology, Chinese Academy of Sciences, Beijing, 100101 China; 7 Savaid Medical School, University of Chinese Academy of Sciences, Beijing, 101408 China

**Keywords:** Ageing, Bone development

## Abstract

Bone homeostasis is maintained by the balance between osteoblastic bone formation and osteoclastic bone resorption. Dysregulation of this process leads to multiple diseases, including osteoporosis. However, the underlying molecular mechanisms are not fully understood. Here, we show that the global and conditional osteoblast knockout of a deubiquitinase *Otub1* result in low bone mass and poor bone strength due to defects in osteogenic differentiation and mineralization. Mechanistically, the stability of FGFR2, a crucial regulator of osteogenesis, is maintained by OTUB1. OTUB1 attenuates the E3 ligase SMURF1-mediated FGFR2 ubiquitination by inhibiting SMURF1’s E2 binding. In the absence of OTUB1, FGFR2 is ubiquitinated excessively by SMURF1, followed by lysosomal degradation. Consistently, adeno-associated virus serotype 9 (AAV9)-delivered FGFR2 in knee joints rescued the bone mass loss in osteoblast-specific *Otub1*-deleted mice. Moreover, *Otub1* mRNA level was significantly downregulated in bones from osteoporotic mice, and restoring OTUB1 levels through an AAV9-delivered system in ovariectomy-induced osteoporotic mice attenuated osteopenia. Taken together, our results suggest that OTUB1 positively regulates osteogenic differentiation and mineralization in bone homeostasis by controlling FGFR2 stability, which provides an optical therapeutic strategy to alleviate osteoporosis.

## Introduction

Bone homeostasis is maintained through the balance between osteoblast-mediated bone formation and osteoclast-mediated bone resorption. Disruption of this process is related to multiple diseases, such as osteoporosis and spontaneous fracture.^1^ Cumulative evidences suggest that bone homeostasis is regulated by cytokines and growth factors, including fibroblast growth factors (FGFs). FGFs bind to the extracellular domain of fibroblast growth factor receptors (FGFR 1–5) and initiate an intracellular transduction cascade, such as the PI3K/AKT, RAS/MAPK, STAT1/p21, JNK, and p38 pathways.^2^ Studies in multiple mouse models and human patients have demonstrated the essential role of FGFRs in skeletal development, congenital bone diseases, and the maintenance of adult bone homeostasis.^3,4^ However, the physiological role of FGF/FGFR signals in different cell types and development stages to regulate bone homeostasis is complicated and requires further exploration.

Ubiquitin system is an enzymatic cascade that conjugant a mono ubiquitin or polyubiquitin chains to target proteins, thereby promoting degradation or changing activities.^5^ Hundreds of ubiquitin-conjugating enzymes (E1, E2, and E3) are responsible for ubiquitin chains formation, while nearly 100 deubiquitinases (DUBs) edit or remove these ubiquitin chains.^6^ Among these DUBs, OTU domain-containing ubiquitin aldehyde-binding proteins Otubain1 (OTUB1) and Otubain2 (OTUB2) are members of the OTU domain family of DUBs.^7^ Previous large-scale studies have indicated the role of OTUB1 in cancer initiation, DNA damage response, neurodegenerative disorders, kidney diseases, and pulmonary fibrosis.^8–13^ Recent studies using mouse models have uncovered the important roles of OTUB1 in immune response and lung development. The deletion of *Otub1* in T or B cells leads to immune cell hyperplasia and autoimmunity.^14,15^ Mice lacking *Otub1* in astrocytes show severe central nervous system autoimmunity, and dendritic cell-specific deletion of *Otub1* impairs the immune response of dendritic cells during infection and inflammation.^16,17^ Moreover, Ruiz-Serrano et al. reported that *Otub1* knockout neonates died quickly after birth due to defects of lung development.^18^ However, the physiological roles and molecular mechanisms of OTUB1 in bone homeostasis remain unknown.

Here, we generated *Otub1* global and conditional knockout mice and uncovered an important role of OTUB1 in bone homeostasis by promoting osteoblast differentiation and mineralization. Moreover, we revealed that the decreased FGFR2 protein levels in *Otub1*-deficient bone tissues is a potential reason for this phenotype. Further studies showed that OTUB1 prevents ubiquitination of FGFR2 catalyzed by the E3 ligase SMURF1, thereby inhibiting FGFR2 lysosomal degradation in osteoblast. Accordingly, osteogenic defects in osteoblast-specific *Otub1*-deleted mice were alleviated by AAV9-delivered FGFR2 in knee joints. Furthermore, OTUB1 expression is downregulated during osteoporosis, and restoring OTUB1 levels attenuates bone loss in an osteoporotic mouse model established by ovariectomy (OVX). In this study, we unveil the positive role of OTUB1 in osteoblast-mediated bone formation by maintaining FGFR2 stability and the osteogenesis effects of OTUB1 provide an optical therapeutic strategy to alleviate osteoporosis.

## Results

### OTUB1 deficiency causes defects in osteogenesis

**Fig. 1:**
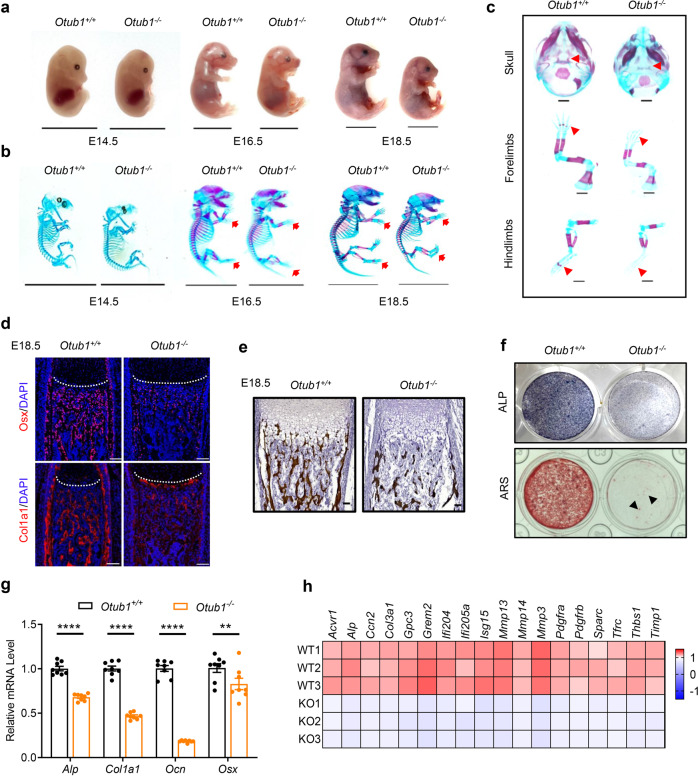
OTUB1 deficiency causes defects in osteogenesis. **a** Representative image of *Otub1*^*+/+*^ and *Otub1*^*−/−*^ embryos at E14.5, E16.5, and E18.5. *n* = 4 per group. Scale bars, 1 cm. **b** Representative images of whole skeleton of *Otub1*^*+/+*^ and *Otub1*^*−/−*^ embryos at E14.5, E16.5, and E18.5. Red arrows indicate the delayed Alizarin red staining in the ribs and phalanges. *n* = 4 per group. Scale bars, 1 cm. **c** Representative images of skeleton of skulls, forelimbs, and hindlimbs in E16.5 *Otub1*^*+/+*^ and *Otub1*^*−/−*^ embryos. Red arrows indicate the delayed Alizarin red staining. *n* = 4 per group. Scale bars, 1 mm. **d** Immunofluorescence assay for expression of osteoblast differentiation markers Osx and Col1a1 in E18.5 *Otub1*^*+/+*^ and *Otub1*^*−/−*^ femurs. *n* = 3 per group. Scale bars, 100 μm. White line indicates the epiphyseal growth plate. **e** Representative images of Von Kossa staining of E18.5 *Otub1*^*+/+*^ and *Otub1*^*−/−*^ mice. *n* = 3 per group. Scale bars, 50 μm. **f** Representative images of ALP and ARS staining of osteoblast cells from E18.5 *Otub1*^*+/+*^ and *Otub1*^*−/−*^ mice after cultured in osteogenic medium for 14 and 28 days. Black arrows indicate calcified nodules. **g** Quantitative RT-PCR analysis of osteogenesis genes mRNA levels in osteoblast cells from *Otub1*^*+/+*^ and *Otub1*^*−/−*^ mice. *n* = 8 per group. **h** Gene expression profiles of the selected osteogenetic genes in osteoblast cells from *Otub1*^*+/+*^ (referred to as WT) and *Otub1*^*−/−*^ (referred to as KO) mice. **p < 0.01, ****p < 0.0001. All data are shown as the mean ± SEM

To determine the physiological role of OTUB1 in bone homeostasis, *Otub1* global knockout mice (referred to as *Otub1*^*−/−*^) were constructed, and their phenotypes were analyzed on embryonic (E) day 14.5 (E14.5), E16.5, E18.5, and postnatal (P) day P0 (Fig. 1a and Supplementary Fig. 1a, b). We observed that *Otub1* knockout significantly decreased the body length at E18.5 and P0 (Fig. 1a and Supplementary Fig. 1c, d). To further investigate the effect of OTUB1 on body length, Alizarin red and Alcian blue double staining of E14.5, E16.5, and E18.5 *Otub1*^*+/+*^ and *Otub1*^*−/−*^ mice were performed. A remarkable delayed bone formation was observed in the skull and limbs of *Otub1*^*−/−*^ mice at E16.5 compared with control littermates (Fig. 1b, c), as well as in the skull, sternum, xiphoid, and phalanges of *Otub1*^*−/−*^ mice at E18.5 (Supplementary Fig. 1e). Because bone formation is associated with osteoblast differentiation and calcification, the expression levels of osteoblast differentiation marker genes and calcification status in *Otub1*^*−/−*^ mice were analyzed. Immunofluorescence staining showed that the expression of early differentiated gene *Osx* and the late differentiated gene *Col1a1* were both decreased in the femurs of E18.5 *Otub1*^*−/−*^ mice (Fig. 1d). Von Kossa staining of the femurs also revealed that the calcification status of ~75% E18.5 *Otub1*^*−/−*^ mice was markedly decreased (Fig. 1e). To further confirm that the osteoblast lineage was specifically affected by OTUB1, we examined chondrocytogenesis and osteoclastogenesis in E18.5 *Otub1*^*−/−*^ mice. Hematoxylin and eosin (H/E) and Safranin O-Fast Green staining of femur sections from E18.5 *Otub1*^*−/−*^ mice showed no significant changes in the morphology and length of the proliferative zone (PZ) and hypertrophic zone (HZ) in the femur growth plate (Supplementary Fig. 1f, g). Similarly, *Otub1* knockout had no effect on osteoclastogenesis (Supplementary Fig. 1h). These data demonstrate that the defective bone formation observed in *Otub1*^*−/−*^ mice was mainly due to defects in the osteoblast lineage.

To further investigate the role of OTUB1 in the osteoblast lineage, we isolated osteoblasts from cranial bones to evaluate the role of OTUB1 in osteogenesis, including proliferation, differentiation, and mineralization. No significant differences in proliferation-related genes, including *Cdkn1a*, *c-Myc*, and *Cyclind1* were found in *Otub1-*deficient osteoblasts (Supplementary Fig. 1i). However, differentiation (measured by ALP staining) and mineralization (measured by ARS staining) were severely disrupted in *Otub1*-deficient osteoblasts (Fig. 1f). Decreased osteogenesis in *Otub1*-deficient osteoblasts was also confirmed by the strongly decreased mRNA levels of an array of osteoblast differentiation marker genes, including *Alp*, *Col1a1, Ocn,* and *Osx* (Fig. 1g). Moreover, mass spectrometry (MS) analysis revealed that the expression levels of multiple osteogenesis-related proteins, such as *ALP*, were downregulated in *Otub1*-deficient osteoblasts (Fig. 1h). Interestingly, mice deficient in OTUB2, a protein with high similarity to OTUB1, were born at the expected Mendelian ratio and appeared phenotypically normal at P0, without obvious bone abnormalities (Supplementary Fig. 1j–l). Together, the delayed bone formation observed in *Otub1* global knockout mice is mainly due to defects in osteoblast differentiation and mineralization.

### Inactivation of OTUB1 in osteoblasts leads to bone mass loss

**Fig. 2:**
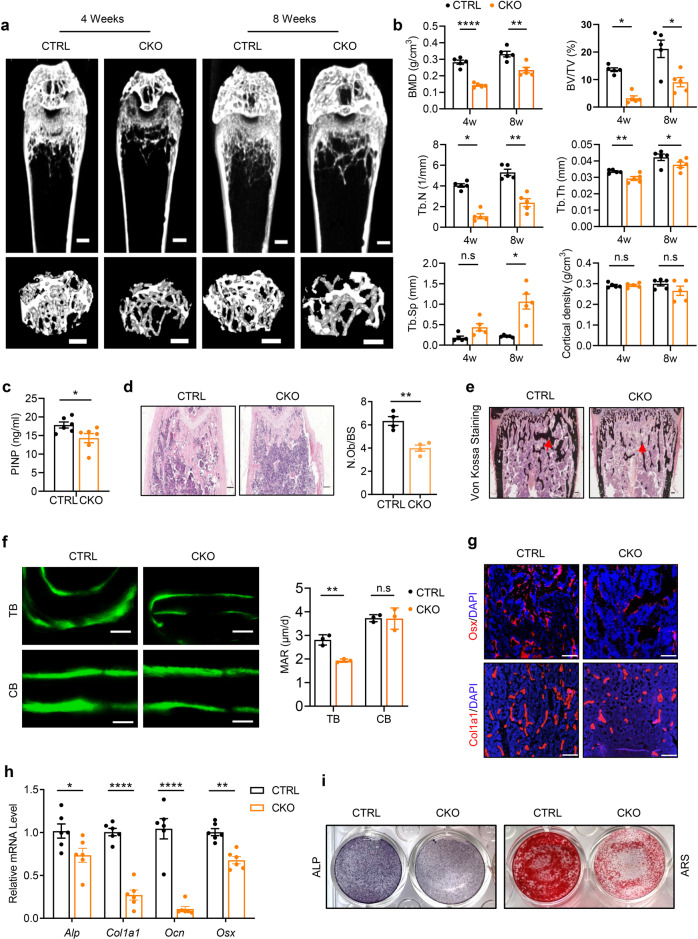
Inactivation of OTUB1 in osteoblast cells leads to bone mass loss. **a** Representative micro-CT images of whole femoral (top) and trabecular (bottom) bones from 4- and 8-week-old male OTUB1 CTRL and CKO mice. *n* = 5 per group. Scale bars, 0.5 mm. **b** Histomorphometric analysis of trabecular bones from (**a**), including bone mass density (BMD), bone volume per tissue volume (BV/TV), trabecular thickness (Tb.Th), trabecular number (Tb.N), trabecular spacing (Tb.Sp) and cortical density. *n* = 5 per group. **c** Serum levels of procollagen type 1 amino-terminal propeptide (P1NP) from 8-week-old male OTUB1 CTRL and CKO mice. *n* = 6 per group. **d** H&E staining and quantitative analysis of osteoblast numbers (N.Ob/BS) in femurs from 8-week-old male *Otub1* CTRL and CKO mice. *n* = 4 per group. Scale bar, 100 μm. **e** Representative images of Von Kossa staining of femurs from 8-week-old male OTUB1 CTRL and CKO mice. *n* = 3 per group. Scale bar, 100 μm. **f** Representative images of calcein double staining and quantitative analysis of mineralization apposition rate (MAR) of the trabecular bones (TB) and cortical bones (CB) from 8-week-old male OTUB1 CTRL and CKO mice. *n* = 3 per group. Scale bar, 20 μm. **g** Immunofluorescence assay for expression of osteoblast-differentiation-related marker genes, including *Osx* and *Col1a1* in OTUB1 CTRL and CKO mice. *n* = 3 per group. Scale bars, 200 μm. **h** Quantitative RT-PCR analysis of osteogenesis genes mRNA levels in 8-week-old male OTUB1 CTRL and CKO mice. *n* = 6 per group. **i** Representative images of ALP and ARS staining of BMSCs from OTUB1 CTRL and CKO mice after cultured in osteogenic medium for 14 and 28 days. *p < 0.05, **p < 0.01, ****p < 0.0001, n.s., not significant. All data are shown as the mean ± SEM

To further confirm the function of OTUB1 in osteoblast lineage cells, we generated an osteoblast-specific *Otub1* deletion mouse model (*Outb1*^*loxP/loxP*^ *Osx-Cre*^*+*^, referred to as CKO) by crossing *Otub1*^*loxP/loxP*^ mice with Osterix-Cre mice (*Osx-Cre*^*+*^). Western blotting and qPCR assays verified that OTUB1 was largely abrogated in bone marrow mesenchymal stem cells (BMSCs) from OTUB1 CKO mice (Supplementary Fig. 2a, b). Osteoblast-specific *Otub1* deletion did not influence birth status or fertility (data not shown). However, immunofluorescence staining of femurs from 4-week-old wild-type mice showed that OTUB1 was enriched in epiphyseal trabecular bones, suggesting its role in linear bone growth (Supplementary Fig. 2c). Indeed, compared with control littermates (*Outb1*^*loxP/wt*^ *Osx-Cre*^*+*^, referred to as CTRL, to exclude the effects of *Osx-Cre*), OTUB1 CKO mice developed marked weight loss and short femoral length independent of sex (Supplementary Fig. 2d). Moreover, the biomechanical properties of these bones were determined via three-point bending, and the results showed that the maximum load and stiffness in the tibia bones of OTUB1 CKO mice were remarkably reduced (Supplementary Fig. 2e). In addition, micro-computed tomography (micro-CT) analysis of femurs from 4- and 8-week-old mice showed that OTUB1 CKO mice began to develop marked bone mass loss after 4 weeks and maintained this severe phenotype until week 8. Both OTUB1 CKO mice had decreased bone mineral density (BMD) and bone volume (BV/TV) in trabecular bones (Fig. 2a, b). Subsequent analysis showed that the reduced trabecular number (Tb.N) was accompanied by reduced trabecular thickness (Tb.Th) and increased trabecular spacing (Tb.Sp) in OTUB1 CKO mice, independent of sex (Fig. 2b and Supplementary Fig. 2f, g). These data demonstrate that inactivation of OTUB1 in osteoblasts leads to low bone mass and poor bone strength.

To unravel whether the low bone mass in OTUB1 CKO mice was due to decreased bone formation, bone formation biomarker N-terminal propeptide of type I procollagen (PINP) was detected. As expected, serum PINP levels were decreased in OTUB1 CKO mice (Fig. 2c). Further histomorphometric analysis revealed that the osteoblast number/bone surface (N.Ob/BS) was significantly decreased in OTUB1 CKO mice (Fig. 2d). Moreover, von Kossa staining showed that the mineralization signal of OTUB1 CKO femurs also decreased (Fig. 2e). In addition, calcein double labeling experiments suggested that the mineralization apposition rate (MAR) was significantly reduced in the trabecular bones of OTUB1 CKO mice compared to that of cortical bones (Fig. 2f). To further confirm that OTUB1 specifically affected the osteoblast lineage, the functions of osteoclasts and chondrocytes were detected. The serum levels of the bone resorption marker C-terminal telopeptide of collagen type 1 (CTX-1) were comparable between OTUB1 CKO and CTRL mice (Supplementary Fig. 2h). Consistently, tartrate-resistant acid phosphatase (TRAP) staining showed that there was no notable difference in the osteoclast surface/bone surface (Oc.S/BS) between OTUB1 CKO and CTRL mice (Supplementary Fig. 2i, j). Similarly, no significant cartilage disorder was observed by Safranin O/Fast green staining in OTUB1 CKO mice (Supplementary Fig. 2k). Together, these results indicate that the low bone mass in OTUB1 CKO mice specifically results from osteogenic defects.

Next, we analyzed the expression of osteogenic markers in femoral bone samples from OTUB1 CTRL and CKO mice in vivo and in vitro. Immunofluorescence staining showed that the expression of *Osx* and *Col1a1* decreased in the femurs of OTUB1 CKO mice (Fig. 2g). Consistently, the expression of marker genes, such as *Alp, Col1a1*, *Ocn,* and *Osx*, was significantly decreased in the femurs of OTUB1 CKO mice (Fig. 2h). Moreover, *Otub1* deletion markedly decreased the differentiation (measured by ALP staining) and mineralization (measured by ARS staining) of the BMSCs (Fig. 2i). Meanwhile, *Otub1* deficiency did not alter osteoblast proliferation because of the comparable expression of proliferation-related marker genes (Supplementary Fig. 2l) and the formation of fibroblast colony-forming unit (CFU-F) (Supplementary Fig. 2m) between OTUB1 CTRL and CKO groups. Collectively, these results indicate that osteoblast-specific OTUB1 is essential for osteogenic differentiation and mineralization.

### OTUB1 stabilizes FGFR2 through inhibiting degradative polyubiquitin chains

**Fig. 3:**
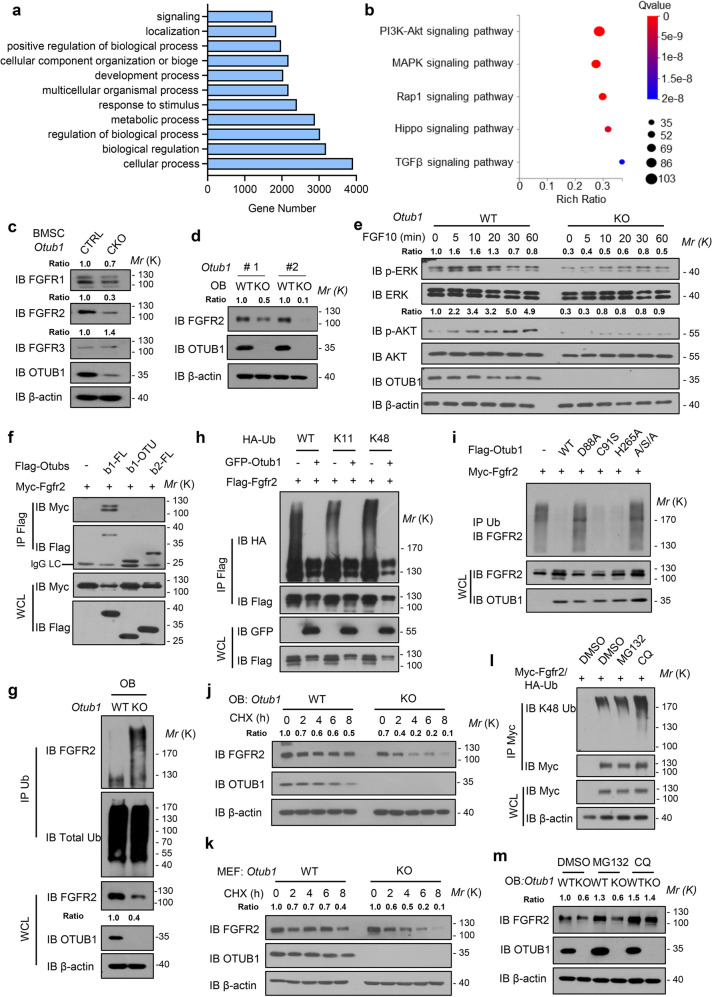
OTUB1 stabilizes FGFR2 through inhibiting degradative polyubiquitin chains. **a** Gene Ontology (GO) enrichment analysis for differentially expressed genes. **b** KEGG enrichment analysis for differentially expressed genes in GO terms regarding development process. **c** Immunoblot of FGFR1, FGFR2 and FGFR3 levels in BMSCs from OTUB1 CTRL and CKO mice. **d** Immunoblot of FGFR2 levels in osteoblasts from *Otub1*^*+/+*^ and *Otub1*^*−/−*^ mice. **e** Immunoblot of the signaling proteins including PI3K and MAPK in *Otub1*^*+/+*^ and *Otub1*^*−/−*^ MEFs with or without 50 ng/mL FGF10 stimulation. **f** Immunoprecipitates of Flag-Otub1-FL, Flag-Otub1-OTU, and Flag-Otub2 transfected with Myc-Fgfr2 and immunoblotted with the indicated antibodies. **g** Immunoblot of FGFR2 polyubiquitination levels in osteoblast cells from *Otub1*^*+/+*^ and *Otub1*^*−/−*^ mice. **h** Immunoprecipitates of FGFR2 in HEK293T cells transfected with HA-tagged ubiquitin with lysine (K) only mutants (All K residues in ubiquitin except the indicated lysine mutated to arginine) and immunoblotted with the indicated antibodies. **i** Immunoprecipitates of FGFR2 in HEK293T cells transfected with Flag-Otub1 or indicated Flag-Otub1 mutants (D88A, C91S, H265A and A/S/A) and immunoblotted with the indicated antibodies. **j** Immunoblot of FGFR2 levels in osteoblast (OB) cells followed by CHX treatment for the indicated periods (left) and quantification of FGFR2 levels in right. **k** Immunoblot of FGFR2 levels in MEF cells followed by CHX treatment for the indicated periods (left) and quantification of FGFR2 levels in right. **l** Immunoblot of FGFR2 polyubiquitination levels in HEK293T cells with the treatments of DMSO, proteasome inhibitor MG132 or the lysosome inhibitor chloroquine (CQ). **m** Immunoblot of FGFR2 levels in *Otub1*^*+/+*^ and *Otub1*^*−/−*^ osteoblast cells with the treatments of DMSO, proteasome inhibitor MG132 or the lysosome inhibitor chloroquine (CQ)

To uncover the potential mechanism of OTUB1 in regulating osteogenesis, we performed RNA-seq using osteoblasts from the cranial bones of *Otub1*^*+/+*^ (WT) and *Otub1*^*−/−*^ (KO) mice. A total of 504 downregulated genes (<0.5-fold) and 301 upregulated genes (>2-fold) genes were identified in OTUB1-deficient osteoblasts (Supplementary Fig. 3a). Next, we used these differentially expressed genes for further gene ontology (GO) analysis (Fig. 3a). Given that OTUB1 was involved in organogenesis, detailed KEGG pathway analysis was focused on GO terms related to the development process. KEGG pathway assessment revealed that PI3K-Akt and MAPK signaling pathways were the top 2 enriched pathway (Fig. 3b). Moreover, UpSet plot of differentially expressed genes from KEGG pathways also showed that PI3K-AKT and MAPK shared 35 genes, indicated that the upstream of them might be regulated by OTUB1 (Supplementary Fig. 3b). Since receptor tyrosine kinases are well characterized to be the upstream of PI3K and MAPK signaling and important for bone development,^19–21^ we enriched the receptor tyrosine kinases-related pathway. Comparative analysis showed that FGF signaling were top 1 enriched signaling among other receptor tyrosine kinases-related pathway (Supplementary Fig. 3c). Therefore, we analyzed the expression of FGFRs in *Otub1* deleted cells. As shown in Fig. 3c, d, among FGFRs, FGFR2 protein levels were significantly decreased. Notably, OTUB1 deficiency did not affect the FGFR2 transcriptional level (Supplementary Fig. 3d). Consistently, the depletion of OTUB1 led to delayed activation of AKT and ERK signaling upon FGFR2 ligand FGF10 stimulation in mouse embryonic fibroblasts (MEFs) (Fig. 3e). It has been reported that OTUB1 regulates neural precursor cell expressed developmentally down‐regulated gene 4‐like (NEDD4L) mediated ubiquitination and degradation of pSMAD2/3,^22^ which is important for osteogenic differentiation.^23^ We next analyzed the expression of pSMAD2/3, pSMAD1/5, SMAD1, and SMAD2/3 in *Otub1*^*+/+*^ and *Otub1*^*−/−*^ osteoblast cells. The results showed that these proteins were not changed in *Otub1*^*−/−*^ osteoblasts, indicated that OTUB1 deletion might not influence the SMAD2/3 and SMAD1/5 signaling during osteoblast development (Supplementary Fig. 3e). Collectively, these results demonstrated FGFR2 was a potential substrate of OTUB1 in osteoblasts.

To further explore the molecular mechanism underlying FGFR2 stability regulated by OTUB1, we assessed the relationship between OTUB1 and FGFR2. Co-immunoprecipitation assays indicated a specific interaction between OTUB1 and FGFR2, but not between the other nine examined members of the OTU family (Supplementary Fig. 3f). Furthermore, this interaction was confirmed using an in vitro GST pull-down assay (Supplementary Fig. 3g). It’s well known that OTUB1 contains two domains: the N-terminal ubiquitin-association (UBA) domain and the C-terminal ovarian tumor (OTU) domain.^7^ Deletion of the UBA domain disrupted the interaction between FGFR2 and OTUB1 (Fig. 3f), indicating that the interaction between OTUB1 and FGFR2 was dependent on the UBA domain of OTUB1, which is absent in OTUB2.

The downregulated protein level of FGFR2 in OTUB1-deficient cells prompted us to test whether OTUB1 is a potential DUB for FGFR2. As shown in Fig. 3g, compared with *Otub1*^*+/+*^ controls, enhanced FGFR2 ubiquitination was detected in *Otub1*^*−/−*^ osteoblasts. Moreover, OTUB1 effectively inhibited degradative Lys11- and Lys48-linked polyubiquitination of FGFR2 (Fig. 3h). It has been reported that OTUB1 either functions as a canonical DUB by directly cleaving ubiquitin chains^24,25^ or inhibits substrate ubiquitination through non-canonically interfering with the functions of E2.^26,27^ To unravel which mechanism is involved in OTUB1-mediated FGFR2 regulation, we mutated E2-binding residue aspartic acid (D) 88, catalytic core residues cysteine (C) 91 and histidine (H) 265 in OTUB1. As shown in Fig. 3i, D88A (aspartic acid mutated to alanine) and ASA (D88A/C91S/H265A), but not C91S (cysteine mutated to serine) and H265A (histidine mutated to alanine), interfered with the ability of OTUB1 to inhibit FGFR2 ubiquitination, implicating an atypical mechanism by which OTUB1 regulates FGFR2 by inhibiting E2 function. Consistent with the effects of OTUB1 on FGFR2 polyubiquitination, protein levels of FGFR2 were dramatically increased upon co-expression with OTUB1 (Supplementary Fig. 3h). Meanwhile, depletion of OTUB1 in osteoblasts and MEFs exerted the opposite effects (Fig. 3j, k).

As previous studies have demonstrated that ubiquitinated receptors could be recruited to the endosomal sorting complex required for transport (ESCRT) complex and transported to multi-vesicular bodies (MVBs) for lysosomal degradation.^28–30^ Therefore, we wondered whether FGFR2 could be degraded by lysosomal pathway. The result revealed that lysosome inhibitor chloroquine (CQ) increased the ubiquitination level of FGFR2 instead of the proteasome inhibitor MG132 (Fig. 3l). Consistently, we found that CQ restored FGFR2 protein levels in OTUB1 KO osteoblasts, whereas MG132 was unable to block the degradation of FGFR2 (Fig. 3m). Together, these data suggest that OTUB1 prevents FGFR2 from lysosomal degradation by inhibiting the degradative polyubiquitin chains.

### OTUB1 cooperates with SMURF1 to regulate the ubiquitination and stability of FGFR2

**Fig. 4:**
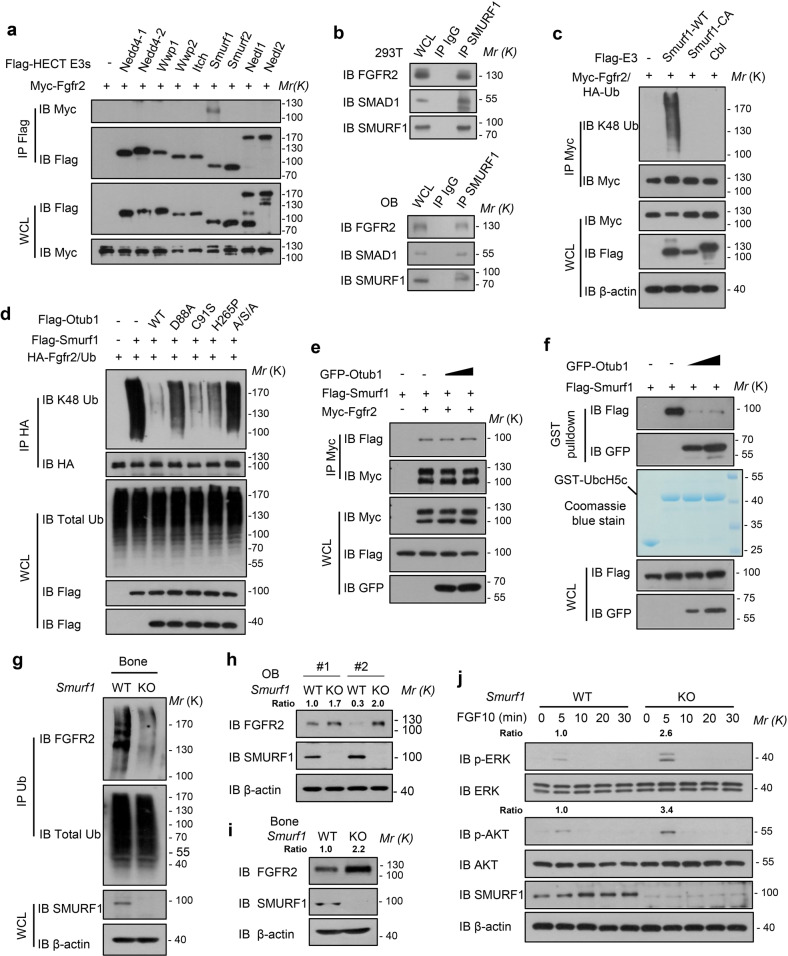
OTUB1 cooperates with SMURF1 to regulate the ubiquitination and stability of FGFR2. **a** Immunoprecipitates of Nedd4 family proteins in HEK293T cells transfected with Myc-Fgfr2 and immunoblotted with the indicated antibodies. **b** Examination of in vivo interaction between SMURF1 and OTUB1 in HEK293T and osteoblast cells. **c** Immunoprecipitates of FGFR2 ubiquitination in HEK293T cells transfected with Flag-Smurf1-WT, Flag-Smurf1-CA, and Flag-Cbl, and immunoblotted with the indicated antibodies. **d** Immunoprecipitates of FGFR2 ubiquitination in HEK293T cells transfected with Flag-Smurf1 or indicated Flag-Otub1 (WT, D88A, C91S, H265A, and A/S/A) and immunoblotted with the indicated antibodies. **e** Immunoblot analysis of the interaction between SMURF1 and its substrate FGFR2 in HEK293T cells with or without GFP-Otub1. **f** Immunoblot analysis of the interaction between SMURF1 and its E2 UbcH5c in vitro. **g** Immunoblot analysis of the ubiquitination of FGFR2 in bones from *Smurf1*^+/+^ and *Smurf1*^−/−^ mice. **h** Immunoblot analysis of FGFR2 expression in *Smurf1*^+/+^ and *Smurf1*^−/−^ osteoblast (OB) cells. **i** Immunoblot analysis of FGFR2 expression in *Smurf1*^+/+^ and *Smurf1*^−/−^ bones. **j** Immunoblot of the signaling proteins including PI3K and MAPK signaling in *Smurf1* WT and KO osteoblast cells with 20 ng/ml FGF10 stimulation

To identify new potential E3 ligases that cooperate with OTUB1 to regulate the ubiquitination of FGFR2, we queried FGFR2 as a substrate in UbiBrowser (http://ubibrowser.ncpsb.org.cn/ubibrowser_v3/).^31^ This bioinformatic analysis suggested that neuronally expressed developmentally downregulated 4 (NEDD4) subfamily members of HECT-type E3 ligases were the significant hits for FGFR2 (Supplementary Fig. 4a). Then, we tested nine members of the NEDD4 subfamily and found that only Smad ubiquitination regulatory factor 1 (SMURF1) specifically interacted with FGFR2 (Fig. 4a). Importantly, endogenous FGFR2 was found to interact with SMURF1 in osteoblasts (OB) and HEK293T cells (Fig. 4b). Domain mapping studies of Smurf1 revealed that the WW and HECT domains were responsible for the FGFR2 interaction (Supplementary Fig. 4b). The E3 ligase Casitas B-lineage lymphoma (CBL), has been reported to regulate FGFRs endocytosis.^32,33^ Thus, we ectopically expressed Smurf1, Cbl, and found that SMURF1 WT (wild-type) specifically increased FGFR2 ubiquitination, but not CBL (Fig. 4c), suggesting that SMURF1 is a potential E3 ligase responsible for FGFR2 stability. Indeed, we found that the exogenous expression of Smurf1 decreased the protein levels of FGFR2, but not Smurf1 CA (catalytic inactivation mutant) (Supplementary Fig. 4c). Meanwhile, CQ restored FGFR2 protein levels in Smurf1 overexpressed HEK293T cells (Supplementary Fig. 4d), suggested that ubiquitination of FGFR2 by SMURF1 underwent lysosomal degradation.

We next performed several experiments to identify how OTUB1 antagonize Smurf1 to maintain the stability of FGFR2. We found that OTUB1-decreased Smurf1-mediated FGFR2 ubiquitination was dependent on the D88 residue (a typical site for E2 binding) (Fig. 4d), in accordance with the results shown in Fig. 3i. It has been reported that OTUB1 inhibits the substrate ubiquitination through interfering with a subfamily of E2 enzymes, such as UbcH5C.^9^ To further elucidate the inhibitory mechanism of OTUB1, the interaction between the E3 Smurf1 and the E2 or the substrate was detected. As shown in Fig. 4e, f, OTUB1 overexpression suppressed the interaction between Smurf1 and E2 UbcH5C, but had no influence on the interaction between Smurf1 and its substrate FGFR2. These data indicated that the OTUB1-E2 interaction disrupted the E3-E2 interaction, thus inhibiting FGFR2 ubiquitination catalyzed by SMURF1.

Previous studies have shown that Smurf1 is a negative regulator in bones.^34–36^ This prompted us to determine the ubiquitination level of FGFR2 in *Smurf1* knockout mouse bones. Compared with the wild-type mice, the ubiquitination of FGFR2 was decreased in the bones of *Smurf1* knockout mice, whereas the ubiquitination of FGFR2 remained unchanged in the spleens (Fig. 4g and Supplementary Fig. 4e). Accordingly, FGFR2 protein level in *Smurf1* knockout osteoblasts and bones were significantly increased, whereas the levels in the brain, liver, and spleen remained unchanged (Fig. 4h, i and Supplementary Fig. 4f). Furthermore, the levels of p-ERK and p-AKT upon FGF10 stimulation were increased in *Smurf1* knockout cells (Fig. 4j), suggesting that SMURF1 is a negative regulator of FGFR2 signaling in bones. Collectively, our data suggest that OTUB1 attenuates SMURF1-mediated FGFR2 ubiquitination by inhibiting the interaction between SMURF1 and E2 UbcH5C in bones.

### FGFR2 rescues OTUB1-deletion-induced bone mass loss and osteogenesis defects

**Fig. 5:**
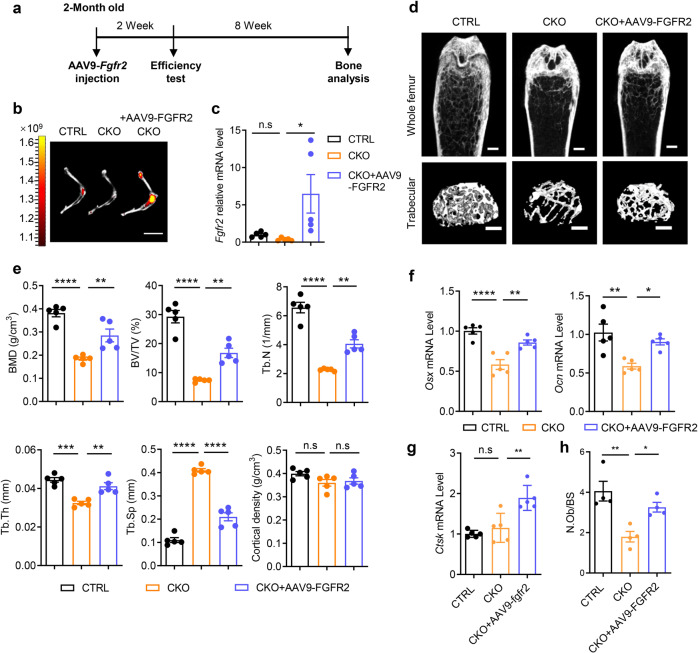
FGFR2 rescues OTUB1 deletion-caused bone mass loss and osteogenesis defects. **a** Schematic representation of experimental design of adeno-associated virus serotype 9 (AAV9)-delivered *Fgfr2* treatment in mice. **b** AAV9-FGFR2 expression in the hindlimb was monitored by IVIS-100 optical imaging 2 months post injection. Scale bars, 1 cm. **c** Quantitative RT-PCR analysis of *Fgfr2* mRNA levels in humeral diaphysis from OTUB1 CTRL, CKO, and CKO with injection of AAV9-FGFR2 groups after 2 months. *n* = 5 per group. **d** Representative micro-CT images of whole femoral (top) and trabecular (bottom) bones from OTUB1 CTRL, CKO, and CKO with injection of AAV9-FGFR2 groups after 2 months. *n* = 5 per group. Scale bars, 0.5 mm. **e** Histomorphometric analysis of trabecular bones in (**d**), including BMD, BV/TV, Tb.Th, Tb.N, Tb.Sp, and cortical density. *n* = 5 per group. **f** Quantitative RT-PCR analysis of osteogenesis genes mRNA levels in femurs from OTUB1 CTRL, CKO, and CKO with injection of AAV9-FGFR2 groups after 2 months. *n* = 5 per group. **g** Quantitative RT-PCR analysis of osteoclastogenesis genes mRNA levels in femurs from OTUB1 CTRL, CKO, and CKO with injection of AAV9-FGFR2 groups after 2 months. *n* = 5 per group. **h** Quantitative analysis of N.Ob/BS in femurs from OTUB1 CTRL, CKO, and CKO with injection of AAV9-FGFR2 groups. *n* = 4 per group. *p < 0.05, **p < 0.01, ***p < 0.001, ****p < 0.0001, n.s., not significant. All data are shown as the mean ± SEM

Adeno-associated virus serotype 9 (AAV9) has been reported to be highly effective for the transduction of osteoblast-lineage cells, including endosteal osteoblasts and osteocytes.^37^ An AAV9 delivery system was used to further verify whether FGFR2 is a bona fide physiological target of OTUB1 in bones. Considering that AAV9-delivered GFP did not affect the bone phenotypes of wild-type mice, AAV9-delivered FGFR2 was injected into the knee joints of two-month-old OTUB1 CKO mice, and the other groups were treated with PBS (Fig. 5a and Supplementary Fig. 5a, b). Two months after injection, the efficiency of AAV9-delivered FGFR2 was examined using IVIS optical imaging and qPCR (Fig. 5b, c). As expected, the decreased bone mass of femurs in OTUB1 CKO mice was largely recovered upon AAV9-FGFR2 injection, along with increased BMD, BV/TV, Tb.N, and Tb.Th, as well as decreased Tb.Sp in the femoral trabecular bone (Fig. 5d, e). Meanwhile, qPCR assays demonstrated that the decreased expression of osteogenic markers such as *Osx* and *Ocn* in OTUB1-CKO tibia bones was normalized with FGFR2 administration (Fig. 5f). In contrast, the expression of osteoclastic markers Cathepsin K (Ctsk) remained unchanged between the OTUB1 CKO and AAV9-FGFR2-treated OTUB1 CKO groups (Fig. 5g). Furthermore, histomorphometric analysis showed that N.Ob/BS was significantly increased in OTUB1 CKO mice with FGFR2 administration compared with those in OTUB1 CKO mice (Fig. 5h). Taken together, these results indicated that FGFR2 is indispensable for osteogenesis in OTUB1-deficient mice.

### OTUB1 overexpression alleviates osteoporosis in OVX mice

**Fig. 6:**
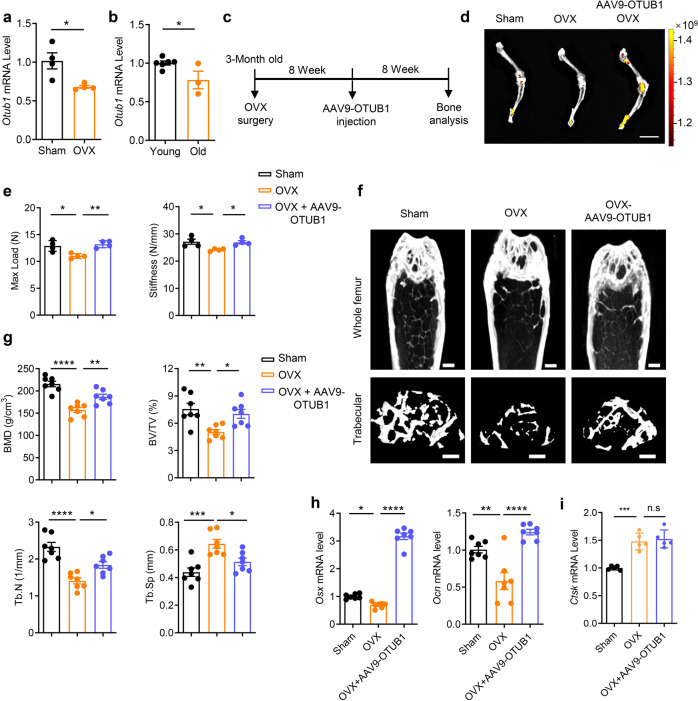
OTUB1 overexpression alleviates osteoporosis in OVX mice. **a** Quantitative RT-PCR analysis of *Otub1* mRNA levels in BMSCs derived from Sham-operated (Sham) and ovariectomy (OVX) mice. *n* = 4 per group. **b** Quantitative RT-PCR analysis of *Otub1* mRNA levels in young (2 months, *n* = 6) and old (2 years, *n* = 3) mice bone tissues. **c** Schematic diagram illustrating the experimental design of AAV9-OTUB1 treatment in OVX mice. Scale bars, 1 cm. **d** AAV9-OTUB1 expression in the hindlimb monitored by IVIS-100 optical imaging 8 weeks post injection. **e** Quantification of maximal loading (Max load) and stiffness of humeral diaphysis from Sham, OVX, and OVX-AAV9-OTUB1 groups. *n* = 4 per groups. **f** Representative micro-CT images of whole femoral (top) and trabecular (bottom) bones from Sham, OVX, and OVX-AAV9-OTUB1 group. *n* = 7 per group. Scale bar, 0.5 mm. **g** micro-CT measurements of BMD, BV/TV, Tb.N, and Tb.Sp in femurs from Sham, OVX, and OVX-AAV9-OTUB1 groups. *n* = 7 per group. **h** Quantitative RT-PCR analysis of osteogenesis gene mRNA levels in femurs from Sham, OVX, and OVX-AAV9-OTUB1 groups. *n* = 7 per group. **i** Quantitative RT-PCR analysis of osteoclastogenesis gene mRNA levels in femurs from Sham, OVX, and OVX-AAV9-OTUB1 groups. *n* = 5 per group. *p < 0.05, **p < 0.01, ***p < 0.001, ****p < 0.0001, n.s., not significant. All data are shown as the mean ± SEM

The positive role of OTUB1 in osteogenesis suggests its potential application in osteoporosis via upregulation of osteogenesis. Based on this, we established an osteoporosis mouse model by ovariectomy (OVX) and found that the mRNA level of *Otub1* in BMSCs derived from OVX mice was markedly decreased compared with the sham-operated (Sham) control (Fig. 6a and Supplementary Fig. 6a, b). Moreover, the mRNA level of *Otub1* was significantly decreased in the bone tissues of aged mice (2 years) compared with that in young mice (2 months) (Fig. 6b). This decreased OTUB1 expression in postmenopausal osteoporosis and age-related osteoporosis prompted us to explore whether the exogenous expression of *Otub1* could alleviate osteoporosis in OVX mice in vivo. AAV9-delivered OTUB1 was injected into the knee joints of OVX mice (Fig. 6c). Two months after surgery, GFP signaling was observed in femurs from AAV9-OTUB1-treated OVX mice, accompanied by increased mRNA level of *Otub1* (Fig. 6d and Supplementary Fig. 6c). Subsequent biomechanical tests revealed that the maximum load and stiffness in tibia bones of AAV9-OTUB1-treated OVX mice were remarkably recovered compared to those in the OVX group (Fig. 6e). Meanwhile, the bone mass of AAV9-OTUB1-treated OVX mice was also significantly restored, along with increased BMD, BV/TV, and Tb.N, and decreased Tb.Sp (Fig. 6f, g). Moreover, the expression of osteogenic markers such as *Osx* and *Ocn* in tibia bones of OVX mice increased with OTUB1 overexpression, while the expression of osteoclastic markers *Ctsk* was comparable in OVX and OTUB1-treated OVX groups (Fig. 6h, i). These results indicated that OTUB1 overexpression in knee joints alleviated osteoporosis of OVX mice by promoting osteogenesis.

## Discussion

The present study demonstrated the crucial role of OTUB1 in osteogenesis (Supplementary Fig. 6d). Homozygous loss of *Otub1* results in impaired bone formation and reduced bone mass owing to defects in osteogenesis. FGFR2, a key modulator of bone development, was responsible for the observed phenotypes in OTUB1-deficient mice. In the absence of OTUB1, FGFR2 ubiquitination catalyzed by Smurf1 results in lysosomal degradation of FGFR2, leading to its compromised downstream signaling. FGFR2 destabilization leads to osteoporosis, which is partially rescued by FGFR2 re-expression. Furthermore, OTUB1 is downregulated during osteoporosis and its overexpression in knee joints alleviates osteoporosis in ovariectomized mice.

Previous studies have shown that OTUB1 plays a crucial role in cancer initiation, DNA damage response, and human diseases such as neurodegenerative disorders, kidney diseases, and pulmonary fibrosis.^9,12,17,38,39^ Our study demonstrated that *Otub1* deficiency leads to delayed bone formation and abnormal osteogenesis in neonates. Moreover, deletion of *Otub1* in osteoblasts causes a reduction in bone mass independent of sex. Further experiments showed that defective differentiation and mineralization occur in *Otub1*-null osteoblasts while their proliferation remained unchanged. Although several studies have revealed the ubiquitin-specific proteases (USPs) family regulate the function of osteoblasts or osteoclasts,^40–43^ our results suggest that OTUB1 is a positive regulator of osteogenesis, which is a previously unidentified important physiological function in the OTU deubiquitinase family.

Osteoporosis is a systemic skeletal disease in the aged population and postmenopausal females, and is characterized by lower bone mass and increased bone fragility.^44^ Local drug delivery systems composed of biomaterials and osteogenic substances are promising strategies for the treatment of osteoporosis. The positive role of OTUB1 in osteogenesis prompted us to examine its potential application in osteoporosis using local AAV9-delivered systems. Our study shows that AAV9-delivered OTUB1 into knee joints prevents bone mass loss in the femurs of postmenopausal female mice. Moreover, the decreased expression of osteogenic markers in the tibia of postmenopausal female mice was restored by OTUB1 administration. These findings support the notion that osteoblast-specific OTUB1 could be used for the treatment of osteoporosis. Recently, a more efficient integrating tetrahedral DNA nanostructure drug delivery system was established, which could be a better carrier to test the effects of osteoblast-specific OTUB1 in the treatment of osteoporosis.^45–47^

Bone homeostasis is regulated by systemic and local release of cytokines and growth factors. FGFR2, a receptor for the cytokine FGF10, is a key regulator of bone development and cancer.^48^ Deletion of *Fgfr2* isoforms results in osteogenesis dysfunction, suggesting its crucial role in bones.^3,49–51^ In this study, we discovered that FGFR2 is a potential substrate of OTUB1. OTUB1 loss results in lower FGFR2 protein levels and subsequently compromised FGFR2 downstream signaling. Moreover, the phenotype of *Otub1*-deleted mice was alleviated by FGFR2 administration in knee joints. FGFR2 signaling is inactivated by a process involving endocytosis and lysosomal degradation.^52–54^ Upon FGFs stimulation, the E3 ubiquitin ligase Cbl forms a ternary complex with phosphor-FRS2 via Grb2, resulting in the ubiquitination of FGFRs and subsequent internalization instead of degradation.^55,56^ We demonstrated that OTUB1 attenuates polyubiquitin chains on FGFR2 independent of its enzyme activity. In the absence of OTUB1, FGFR2 is excessively ubiquitinated by SMURF1 and undergoes lysosomal degradation, which is inhibited by the lysosomal inhibitor CQ. Our results indicate an alternative lysosomal degradation pathway for FGFR2, mediated by the newly discovered E3 Smurf1 instead of Cbl. Taken together, our data indicate that OTUB1 prevents FGFR2 from lysosomal degradation by inhibiting the interaction between the E3 ligase Smurf1 and E2 UbcH5C in osteoblast cells.

In conclusion, our study reveals that OTUB1 cooperates with Smurf1 to regulate osteogenesis by specifically editing the ubiquitination of FGFR2. Excessive ubiquitination leads to lower FGFR2 protein levels and compromised downstream signaling, ultimately causing anostosis in *Otub1*-deficient mice, which is partially rescued by re-expression of FGFR2. Moreover, OTUB1 acts as a positive regulator of osteogenesis to prevent bone mass loss in an osteoporotic mouse model established by ovariectomy. Our study provides new insights into the physiological function of OTUB1 in osteogenesis and indicates that targeting osteoblast-specific OTUB1 is a potential therapeutic strategy in osteoporosis.

## Materials and methods

### Mouse models

All experimental procedures were approved by the Institutional Animal Care and Use Committee of the Beijing Institute of Lifeomics (IACUC-DWZX-2020-663). All mice were maintained on a C57BL/6J background and housed under standard pathogen-free conditions. *Otub1*^*loxP/loxP*^ mice were generated by Gempharmatech Co., Ltd. (XM000975) with *LoxP* sites flanking exons 2 and 3 of *Otub1*. *Otub1*^*loxP/loxP*^ mice were crossed with transgenic mice expressing Cre recombinase purchased from JAX (JAX 006054) to generate *Otub1*^*−/−*^ mice. *OSX-Cre* mice were a gift from Prof. Weiguo Zou (Shanghai Institute of Biochemistry and Cell Biology, CAS) and crossed with *Otub1*^*loxP/loxP*^ mice to generate pre-osteoblast cell-specific knockout mice. *Otub2*^*−/−*^ mice were generated by crossing *Otub2*^*loxP/loxP*^ mice (XM000974) with transgenic mice expressing Cre recombinase, purchased from JAX.

### Antibodies and plasmids

The following antibodies were used: anti-OTUB1 (ab175200), anti-OTUB1 (ab198214, for IHC), anti-Smurf1 (ab57573), anti-RunX2 (ab192256), and anti-OSX (ab209484), purchased from Abcam. Anti-OTUB2 antibody (GTX83953) was purchased from GeneTex. Anti-FGFR1 (9740), anti-FGFR2 (23328), anti-FGFR3 (4574), anti-AKT (4691), anti-p-Ser473-AKT (4060), anti-p-Ser308-AKT (13038), anti-ERK1/2 (4695), and anti-p-Thr202/Tyr204-ERK1/2 (4370) antibodies were purchased from Cell Signaling Technology. Anti-GAPDH (sc-365062) and anti-GST (sc-374171) antibodies were purchased from Santa Cruz Biotechnology. Anti-Myc (M047-3), anti-Flag (M185-3), anti-HA (M180-3), and anti-Multi Ubiquitin (D058-3) antibodies were purchased from MBL. Anti-β-actin (AC026) and anti-COL1A1 (A16891) antibodies were purchased from Abclonal. Peroxidase-AffiniPure goat anti-rabbit IgG (111-035-003) and Peroxidase-AffiniPure goat anti-mouse IgG (115-035-003) were purchased from Jackson.

Full-length OTUB1 WT, D88A, C91S, H265A, ASA mutants, and Flag-HECT E3s were cloned into the pFlag-CMV-2 vector. Full-length FGFR2 was cloned into the pEF6/Myc-His vector.

### Cell transfection and immunoprecipitation

HEK293T cells were transfected with the corresponding expression plasmids using LipoPlus (Genestar, C101-01). After 48 h, the cells were lysed with HEPES lysis buffer (20 mM HEPES (pH 7.2), 50 mM NaCl, 0.5% Triton X-100, 1 mM NaF, and 1 mM dithiothreitol) containing protease inhibitors (Roche, 11697498001). After centrifugation at 12,000 × *g* for 10 min at 4 °C, the supernatant was incubated with the corresponding primary antibody and protein A/G agarose beads (Santa Cruz, sc-2003) at 4 °C overnight. The beads were washed three times with HEPES buffer and boiled for western blotting. Results were measured and analyzed using the ImageJ software.

For ubiquitination, cells were treated with 20 μM of the proteasome inhibitor MG132 (Sigma-Aldrich, M8699) for 8 h before sample collection. Cells or tissues were lysed in RIPA lysis buffer (50 mM Tris (pH 7.5), 150 mM NaCl, 1% NP-40, 10 mM NaF, and 1 mM Na_3_VO_4_) supplemented with protease inhibitor (Roche, 11697498001). The immunocomplexes were washed several times with lysis buffer, resolved by SDS-PAGE, and immunoblotted with the indicated antibodies.

### Protein half-life assay

Cells were treated with cycloheximide (MedChemExpress, HY-12320) for the indicated times, lysed, and subjected to western blotting.

### GST pull-down

GST-OTUB1 was purified from BL21 and bound to glutathione Sepharose 4 B beads (GE), followed by incubation in bone lysates at 4 °C overnight. After washing four times with GST binding buffer (100 mM NaCl, 10 mM Tris, 50 mM NaF, 2 mM EDTA, 0.5 mM Na_3_VO_4_, and 1% NP-40), the beads were boiled and analyzed by western blotting.

### RNA extraction and qPCR

Total RNA was extracted from cells or bones with TRIzol reagent (Invitrogen, 10296010). For bone samples, a self-designed grinder was used to adequately broke bones samples. The bone powder was harvested and resuspended in 1 mL of TRIzol and lysed at room temperature (RT) for 5 min. Then, 0.2 mL of chloroform (Sinopharm, 1006818) was added, and the solution was thoroughly mixed and incubated at RT for 5 min. Next, the mixture was centrifuged at 12,000 × *g* for 15 min at 4 °C. The collected supernatant was mixed with 0.5 ml isopropanol (Sinopharm, 80109296) at RT for 5 min to extract RNA. RNA pellets were harvested by centrifugation (7000 × *g* and 4 °C for 15 min) and washed twice with 75% ethanol. Finally, The RNA pellet was dissolved in 50 μL of nuclease-free water at 60 °C for 10 min.

One microgram of RNA was used for reverse transcription using ReverTra Ace qPCR RT Master Mix (Toyobo, FSQ-201). Real-time PCR was performed on a LightCycler 96 (Roche) using 2X RealStar SYBR Mixture (Genestar, A301). The relative gene expression normalized to that of GAPDH was calculated using the 2^−ΔΔCt^ method. Primer sequences used in this study are listed in Supplementary Table 1.

### Isolation of mouse osteoblasts cells and BMSCs

Osteoblasts were isolated from the calvaria of E18.5 neonates using 0.1% collagenase type I (Sigma-Aldrich, C0130), and were cultured in α-Minimum Essential Medium (α-MEM) (Gibco) containing 10% fetal bovine serum (GEMINI, 900-108) and penicillin-streptomycin solution (CellWorld, C0160-611).

BMSCs were flushed from 8-week-old mice using fresh α-MEM. The cells were seeded in 60-mm plates for 48 h. After washing with PBS to remove non-adherent cells, adherent cells were cultured in a fresh medium. Cells from passage two to passage five were used for the experiments.

### RNA-seq analysis

Osteoblasts from *Otub1*^*+/+*^ and *Otub1*^*−/−*^ mice (*n* = 3 per group) were prepared for RNA sequencing according to the protocol of the Beijing Genomics Institute. RNA-seq analysis was performed using the Dr. Tom Multi-Omics Data Mining System (https://biosys.bgi.com).

### In vitro osteoblastic differentiation and mineralization

To examine osteogenic differentiation, mouse osteoblasts, and 2.5 × 10^5^ BMSCs were seeded in 12-well plates and cultured in osteogenic differentiation basal medium (Cyagen, MUXMT-03021-175). The medium was replaced every two or three days. At days 14 and 28 after incubation, cells were fixed with 4% paraformaldehyde (PFA) and then stained using an ALP detection kit (Beyotime, C3206) and 1% Alizarin red S (Solarbio, G1452) according to the manufacturer’s instructions.

### CFU-F assays

BMSCs (2 × 10^5^) from OTUB1 CTRL and CKO mice were seeded in a 12-well plate and cultured in α-MEM. On day 14, the cells were fixed with 4% PFA and stained with 1% Crystal Violet Ammonium Oxalate Solution (Solarbio, G1062).

### Whole-mount skeletal staining

Mouse skins and adipose tissues were carefully removed using forceps, and the remaining bodies were fixed in 95% ethanol overnight. After processing with acetone overnight to permeabilize cell membranes and dissolve fat, the cartilage was stained with Alcian blue 8GX (Solarbio, G8140) for 24 h, and then decolorized in 70% ethanol three times and 95% ethanol overnight. After being cleared in 1% KOH for 1 h, the bodies were immersed in Alizarin Red S (Solarbio, G8550) solution for 3–4 h to counterstain the bone. After clearing in a 50% glycerol:50% (1%) KOH solution, the skeletons were stored in 100% glycerol.

### Immunohistochemical staining and immunofluorescence

Bone samples were dissected and fixed in 4% PFA for 48 h. For embryonic mice, bone tissues were decalcified in 0.5 M EDTA for 2 days. For postnatal mice, bone tissues were decalcified for 7 days. The specimens were then cut into 5-μm-thick sections. After deparaffinized and rehydrated, bone sections were then incubated in hematoxylin and eosin staining solution (Solarbio, G1120), TRAP staining solution (Solarbio, G1492) and Safranin O/Fast green staining solution (Solarbio, G1371) according to the manufacturer’s instructions. Bone static histomorphometric analyses for osteoblast numbers/bone surface (N.Ob/BS) and osteoclast surface/bone surface (Oc.S/BS) were performed using the Bioquant Osteo software (Bioquant Image Analysis Corp, USA).

For immunofluorescence, hydrated bone sections were treated with sodium citrate antigen retrieval solution (ZSGB-BIO, ZLI-9064) at 72 °C for 6 h, and then incubated in blocking solution (ZSGB-BIO, ZLI-9022) for 20 min at room temperature. Primary antibodies were diluted in antibody dilution buffer (ZSGB-BIO, ZLI-9030) and used for overnight staining at 4 °C. Next, sections were incubated with horseradish peroxidase-conjugated goat anti-rabbit IgG polymer reagent (Vector, 30125) for 30 min at 37 °C. Immunoreactivity was visualized using cyanine 3 (Cy3), according to standard procedures.

### Von Kossa staining

For the embryonic mice, undecalcified bone specimens were dehydrated and embedded in paraffin. Then, 5-μm sections were cut, conventionally dewaxed, and hydrated. The sections were then stained with von Kossa Silver Solution (Solarbio, G3282) under UV light for 30 min. The mixture was rinsed with distilled water and treated with Hypo Solution for 2 min. Finally, the material was dehydrated, clarified, and sealed with resin. For postnatal mice, the fixed femurs were embedded in methylmethacrylate (Servicebio, GP1134). The embedding agent was then removed, and the femurs were rehydrated in distilled water. Von Kossa staining was performed according to the protocols provided by Servicebio (G1043).

### Calcein double labeling

Mice were injected intraperitoneally with calcein (20 mg/kg) 3 and 10 d before skeleton collection. Femurs were fixed in 4% PFA and dehydrated in 30% sucrose at 4 °C overnight. Non-demineralized femurs were embedded using an optimal cutting temperature compound and cut into 10 μm. Fluorescence signals were captured using a Pannoramic MIDI scanner (3DHISTECH, Hungary). Histomorphometric analysis of the mineral apposition rate (MAR) was performed using ImageJ software.

### ELISA analysis

Plasma samples were obtained and centrifuged at 3000 rpm for 10 min at 4 °C before being processed. Quantitative determination of CTX1 (Cloud-Clone, CEA665Mu) and PINP (Cloud-Clone, CEA957Mu) was performed using a kit according to the manufacturer’s instructions.

### Micro CT analysis

Mouse femurs were fixed in 4% PFA for 48 h and then stored in 70% ethanol at 4 °C before scanning. Images of the femurs were scanned using the Inveon MM system (Siemens, Germany) with a spatial resolution of 9.2 μm and setting at 60 kV and 220 μA. Three-dimensional reconstruction of trabecular and cortical bone was generated with COBRA Exxim (EXXIM Computing Corp, Livermore, CA) software from the lowest end of the growth plate extended by 0.5 mm. The trabecular and cortical parameters were assessed using Inveon Research Workplace (Siemens, Munich, Germany) analysis software.

### Biomechanical test

Immediately after dissection, the tibias were subjected to a three-point bending test using a universal testing device (CellScale Biomaterials Testing, Waterloo, ON, Canada). Biomechanical measurement data were collected from the load-deformation curves. The maximum load (N) was recorded, and the slope of the linear portion that represents the material elasticity was recorded to calculate the stiffness (N/mm).

### AAV9 injection

AAV9 vectors harboring *Fgfr2* (AAV9-FGFR2) and *Otub1* (AAV9-OTUB1) were prepared by GENECHEM (Shanghai, China). A single dose of 2 × 10^10^ virus was injected into osteoblast-specific *Otub1*-deleted male mice (8 weeks old) and OVX mice (8 weeks after surgery) via the joint cavity. Eight weeks after the operation, all mice were sacrificed and bone marrow tissues were collected for further analysis.

### Statistical analysis

Statistical analyses were performed using GraphPad software (version 8.0.2). Statistical significance was calculated using two-tailed Student’s t-test or one-way analysis of variance (ANOVA). *p* < 0.05 was considered statistically significant.

## Supplementary information

Supplementary Materials

Unprocessed western blot data

## Data Availability

Raw protein differential profiling data have been deposited to the ProteomeXchange Consortium (http://proteomecentral.proteomexchange.org) via the iProX partner repository^57^ with the dataset identifier PXD039507. The original RNA-seq data of *Otub1*^*+/+*^ and *Otub1*^*−/−*^ osteoblast cells have been deposited in the database of the Sequence ReadArchive (SRA) (https://www.ncbi.nlm.nih.gov/bioproject/) under the accession number PRJNA924753. All the other data generated for this study are available from the corresponding authors upon reasonable request.
