# Supplementary material for: OTUB1 promotes osteoblastic bone formation through stabilizing FGFR2
Source: Signal Transduct Target Ther. 2023 Apr 7;8:142. doi: 10.1038/s41392-023-01354-2 (PMC10079838; doi:10.1038/s41392-023-01354-2)
Supplement: Supplementary file 1 — Supplementary Materials [file 41392_2023_1354_MOESM1_ESM.docx]

Supplementary Materials for

**OTUB1 promotes osteoblastic bone formation through stabilizing FGFR2**

Qiong Zhu^#^, Yesheng Fu^#^, Chun-Ping Cui, Yi Ding, Zhikang Deng, Chao Ning, Fan Hu,

Chen Qiu, Biyue Yu, Xuemei Zhou, Guan Yang, Jiang Peng, Weiguo Zou, Cui Hua Liu^*^,

Lingqiang Zhang^*^

Correspondence to: zhanglq@nic.bmi.ac.cn (L.Z.) & liucuihua@im.ac.cn (C.H.L.)

**This PDF file includes:**

Figures. S1 to S6

Table S1

#
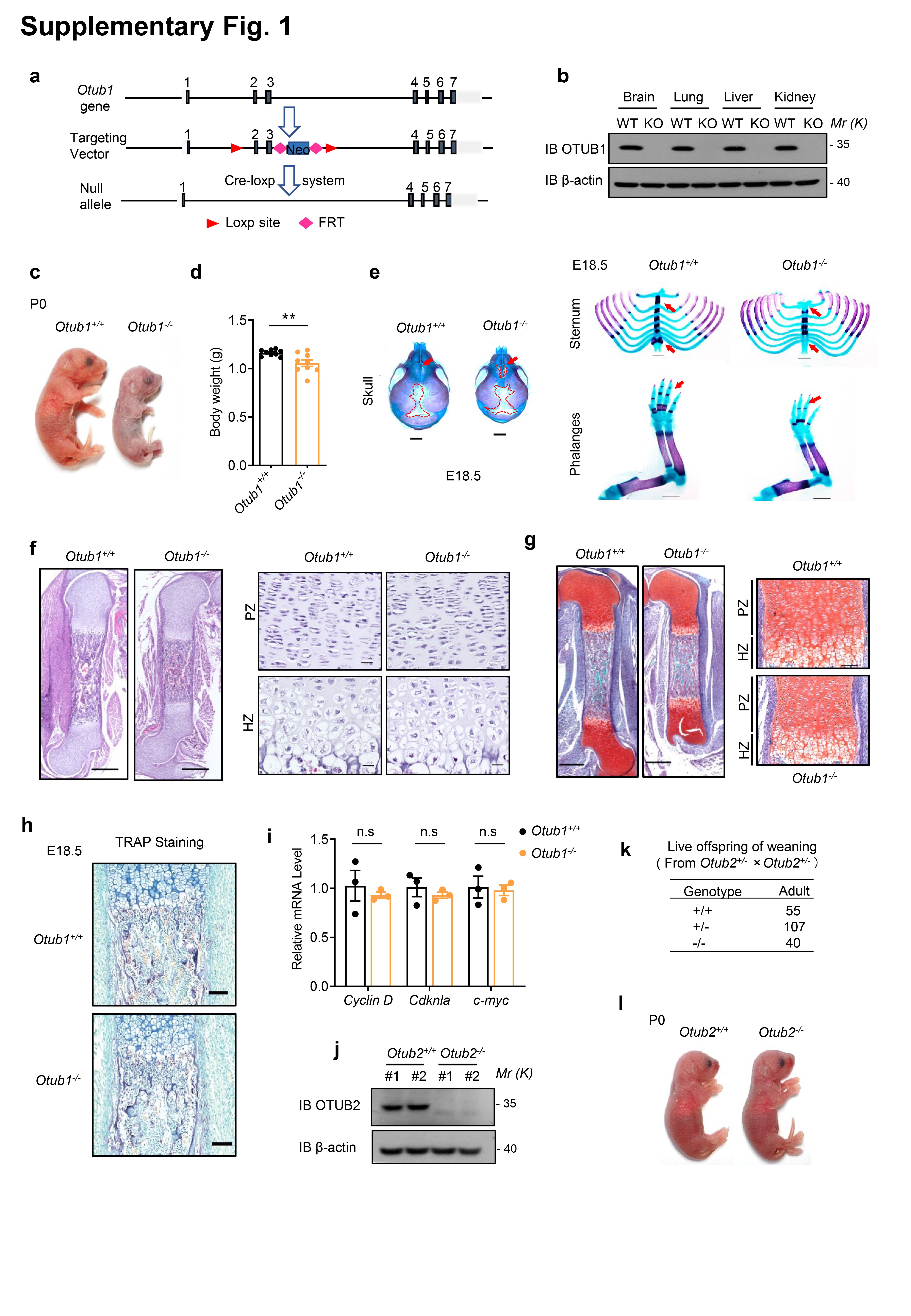
Supplementary information

**Fig. S1. Characterization of bone phenotypes in *Otub1^-/-^* mice.**

**a** Diagram of *Otub1* knockout strategy. The black boxes indicate *Otub1* exons, and the red triangles indicate *loxP* sites.

**b** Immunoblot analysis of OTUB1 protein levels in *Otub1^+/+^* and *Otub1^-/-^* brains, lungs, livers and kidneys at E18.5.

**c** Representative images of P0 *Otub1^+/+^* and *Otub1^-/-^* neonates.

**d** Quantification of body weight from *Otub1^+/+^* and *Otub1^-/-^* mice at E18.5. n = 9 per group.

**e** Representative images of skeleton of skulls, sternums and phalanges in E18.5 *Otub1^+/+^* and *Otub1^-/-^* embryos. Red arrows indicate the delayed Alizarin red staining. n = 3 per group. Scale bars, 1 mm.

**f** Hematoxylin and eosin (H/E) staining of femurs from E18.5 *Otub1^+/+^* and *Otub1^-/-^* embryos. n = 3 per group. Scale bars, 0.5 mm. Zoomed-in image of proliferative zone (PZ) and hypertrophic zone (HZ) are shown in left. Scale bars, 10 μm.

**g** Safranin O/Fast green staining of femur sections from E18.5 *Otub1^+/+^* and *Otub1^-/-^* mice. n = 3 per group. Scale bars, 0.5 mm. Zoomed-in image of proliferative zone (PZ) and hypertrophic zone (HZ) are shown in left. Scale bars, 50 μm.

**h** TRAP staining of femur sections from E18.5 *Otub1^+/+^* and *Otub1^-/-^* mice. n = 3 per group. Scale bar, 100 μm.

**i** Quantitative RT-PCR analysis of proliferation-related genes mRNA levels in osteoblast cells from *Otub1^+/+^* and *Otub1^-/-^* mice. n = 3 per group.

**j** Immunoblot analysis of OTUB2 protein levels in *Otub2^+/+^* and *Otub2^-/-^* lung at E18.5. **k** Mendelian frequencies obtained from intercrossing *Otub2^+/-^* mice.

**l** Representative images of P0 *Otub2^+/+^* and *Otub2^-/-^* mice.

***p* < 0.01, n.s., not significant. All data are shown as the mean ± SEM.


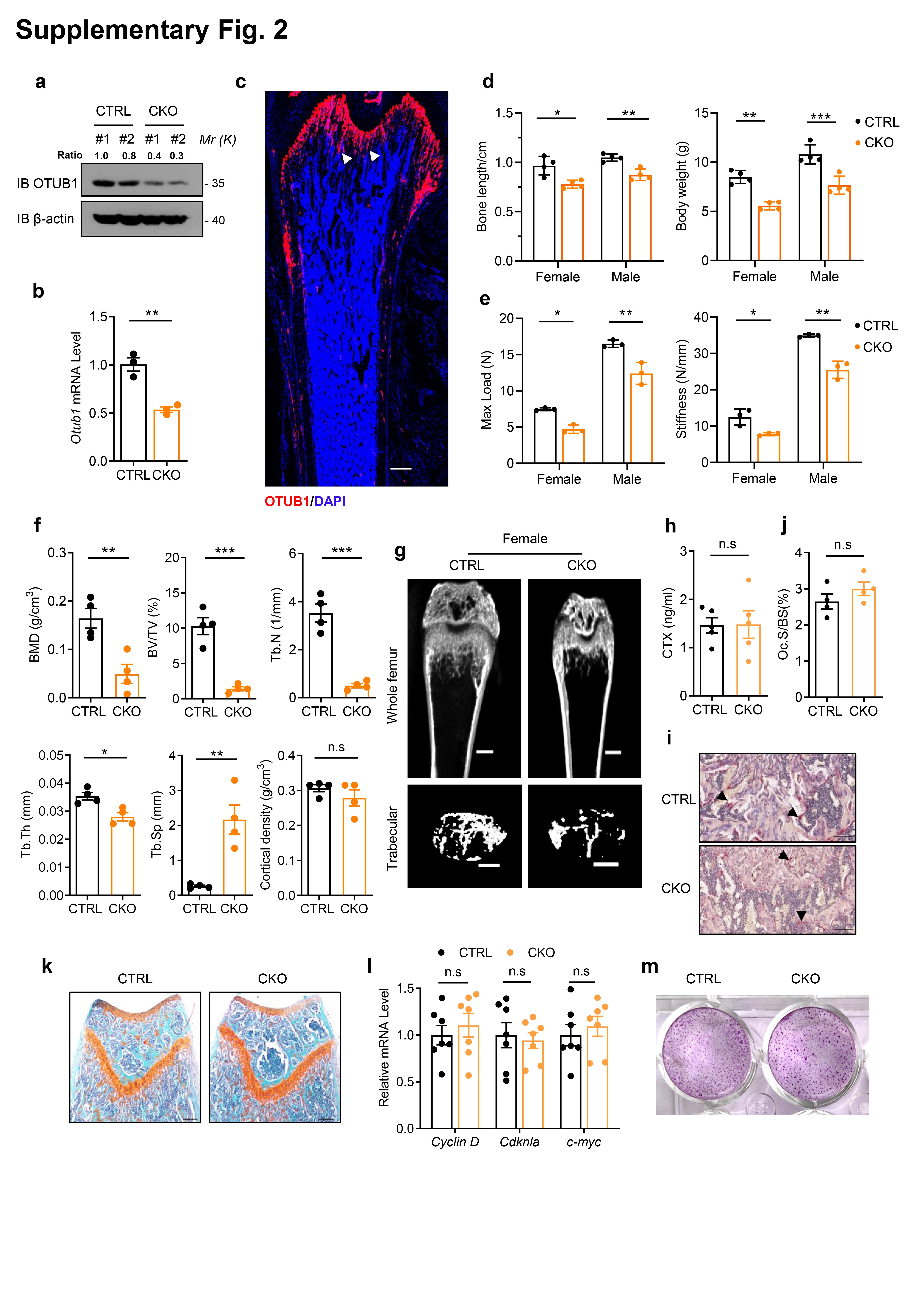


**Figure. S2 Phenotypes of OTUB1 CTRL and CKO mice.**

**a, b** Quantitative analysis of the knockout efficiency of OTUB1 in protein (**a**) and mRNA levels (**b**) in BMSCs from 8-week-old female OTUB1 CTRL and CKO mice.

**c** Immunofluorescence analysis of OTUB1 expression in 4-week-old femurs from wildtype mice. Scale bar, 0.5 mm.

**d** Quantification of femur lengths (left) and body weights (right) of 4-week-old male and female OTUB1 CTRL and CKO mice. n = 4 per group.

**e** Quantification of maximal loading (Max Load) and stiffness of humeral diaphysis from 4-week-old male and female OTUB1 CTRL and CKO mice. n = 3 per group.

**f** Histomorphometric analysis of trabecular bones from 8-week-old female OTUB1 CTRL and CKO mice, including BMD, BV/TV, Tb.Th, Tb.N, Tb.Sp and cortical

density. n = 4 per group.

**g** Representative micro-CT images of whole femoral (top) and trabecular (bottom) bones from 8-week-old female OTUB1 CTRL and CKO mice. n = 4 per group. Scale bars, 0.5 mm.

**h** Serum levels of bone resorption marker C-terminal telopeptide of collagen type 1 (CTX-1) from 8-week-old male OTUB1 CTRL and CKO mice. n = 4 per group.

**i, j** Representative images of TRAP staining (**i**) and quantitative analysis of osteoclast surface/bone surface (Oc.S/BS) (**j**) of femurs from 8-week-old male OTUB1 CTRL and CKO mice (n = 4 per group). Scale bar, 100 μm.

**k** Safranin O/Fast green staining of femur sections from 8-week-old male OTUB1 CTRL and CKO mice. n = 3 per group. Scale bars, 0.5 mm.

**l** Quantitative RT-PCR analysis of proliferation-related genes mRNA levels in BMSCs from OTUB1 CTRL and CKO mice. n = 7 per group.

**m** Representative image of crystal violet staining of cell proliferation assay. **p* < 0.05, ***p* < 0.01, ****p* < 0.001, n.s., not significant. All data are shown as the mean ± SEM.


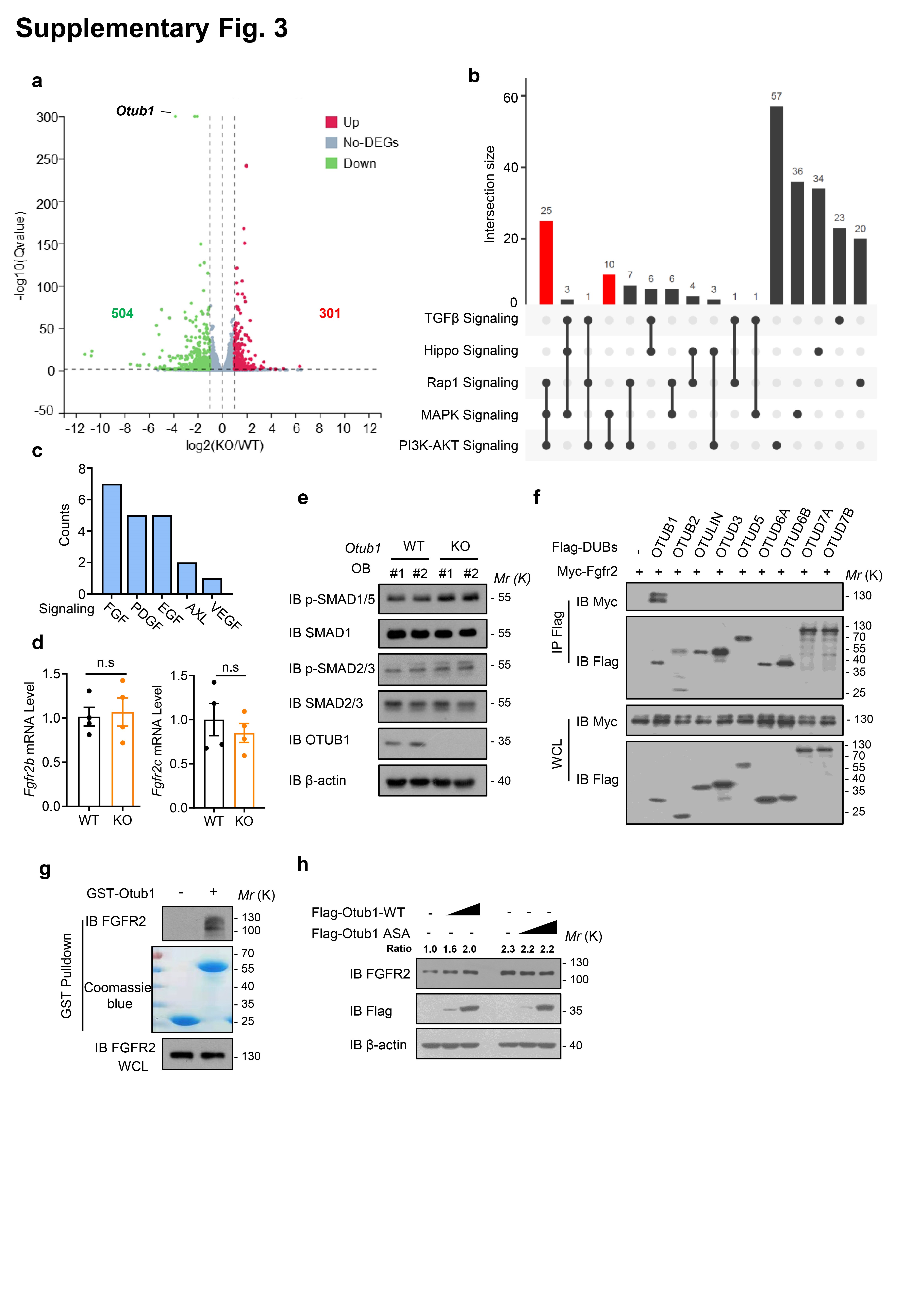


**Figure. S3 OTUB1 targets FGFR2 in FGF signaling.**

**a** Differentially expressed genes in *Otub1^+/+^* and *Otub1^-/-^* osteoblast cells plotted as a volcano plot.

**b** UpSet plot of differentially expressed genes from top 5 KEGG pathways.

**c** Enrichment analysis of top 5 RTK signaling from differentially expressed genes.

**d** Quantitative RT-PCR analysis of *Fgfr2b* and *Fgfr2c* mRNA levels in osteoblast cells from *Otub1^+/+^* and *Otub1^-/-^* mice. n = 4 per group.

**e** Immunoblot of pSMAD2/3, pSMAD1/5, SMAD1 and SMAD2/3 in *Otub1^+/+^* and *Otub1^-/-^* osteoblast cells.

**f** Immunoprecipitates of OTU deubiquitinases in HEK293T cells transfected with Myc-Fgfr2 and immunoblotted with the indicated antibodies.

**g** *In vitro* binding assay of purified GST-OTUB1 and whole lysates was performed, and the reaction mixture was immunoblotted with the indicated antibodies.

**h** Immunoblot of FGFR2 levels in HEK293T cells transfected with Flag-Otub1-WT and Flag-Otub1-ASA.

n.s., not significant. All data are shown as the mean ± SEM.


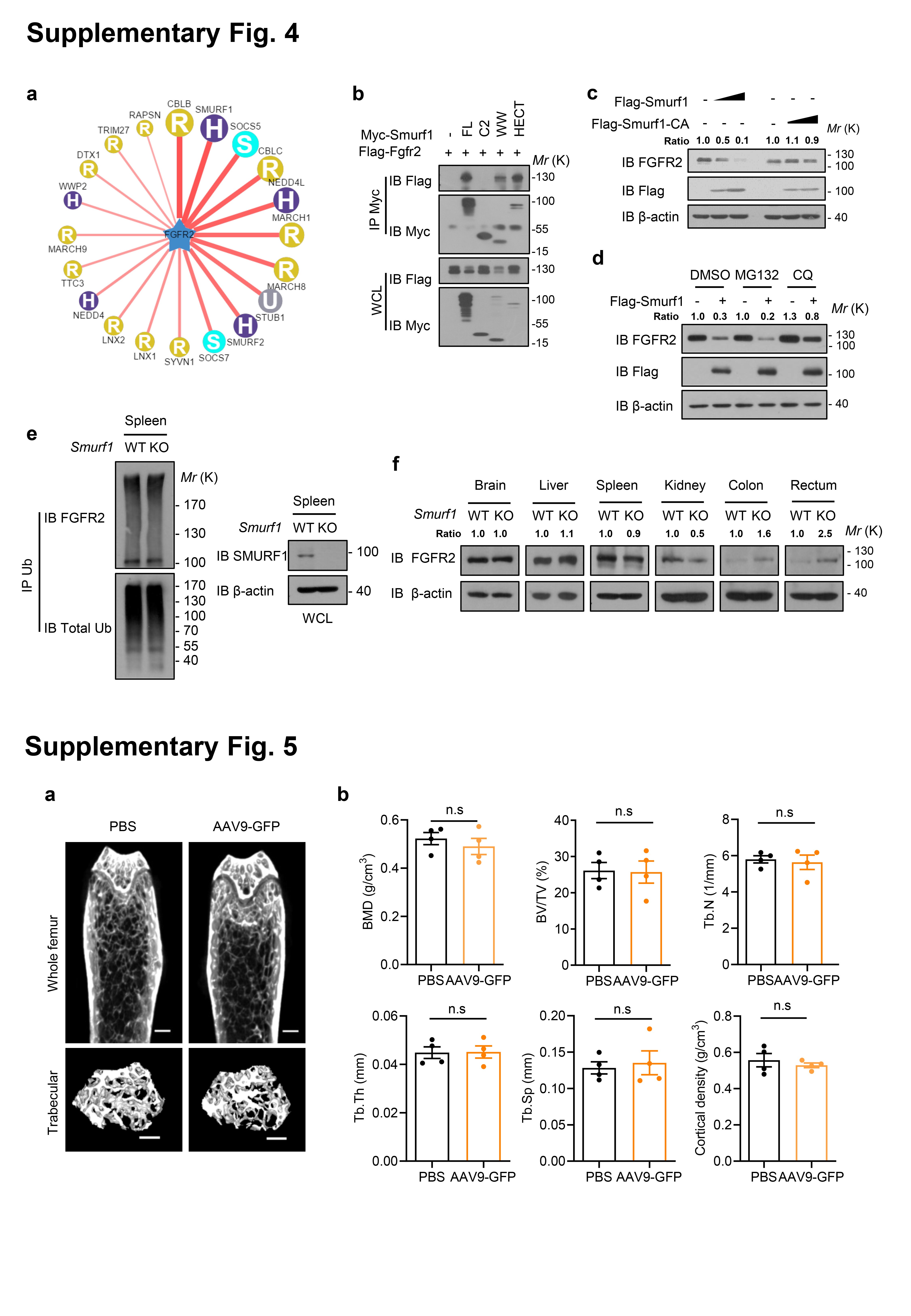


**Figure. S4. SMURF1 decreases FGFR2 stability by interacting with FGFR2.**

**a** Schematic representation of potential interaction of E3s with FGFR2 by using UbiBrowser 1.0.

**b** Immunoblot analysis the domain of SMURF1 that interacts with OTUB1.

**c** Immunoblot of FGFR2 levels in HEK293T cells transfected with Flag-Smurf1-WT and Flag-Smurf1-CA.

**d** Immunoblot of FGFR2 levels with DMSO, proteasome inhibitor MG132 and the lysosome inhibitor chloroquine (CQ) in HEK293T cells transfected with Flag-Smurf1.

**e** Immunoblot analysis of FGFR2 ubiquitination in *Smurf1^+/+^* and *Smurf1^-/-^* spleens.

**f** Immunoblot analysis of FGFR2 protein levels in *Smurf1^+/+^* and *Smurf1^-/-^* tissues.


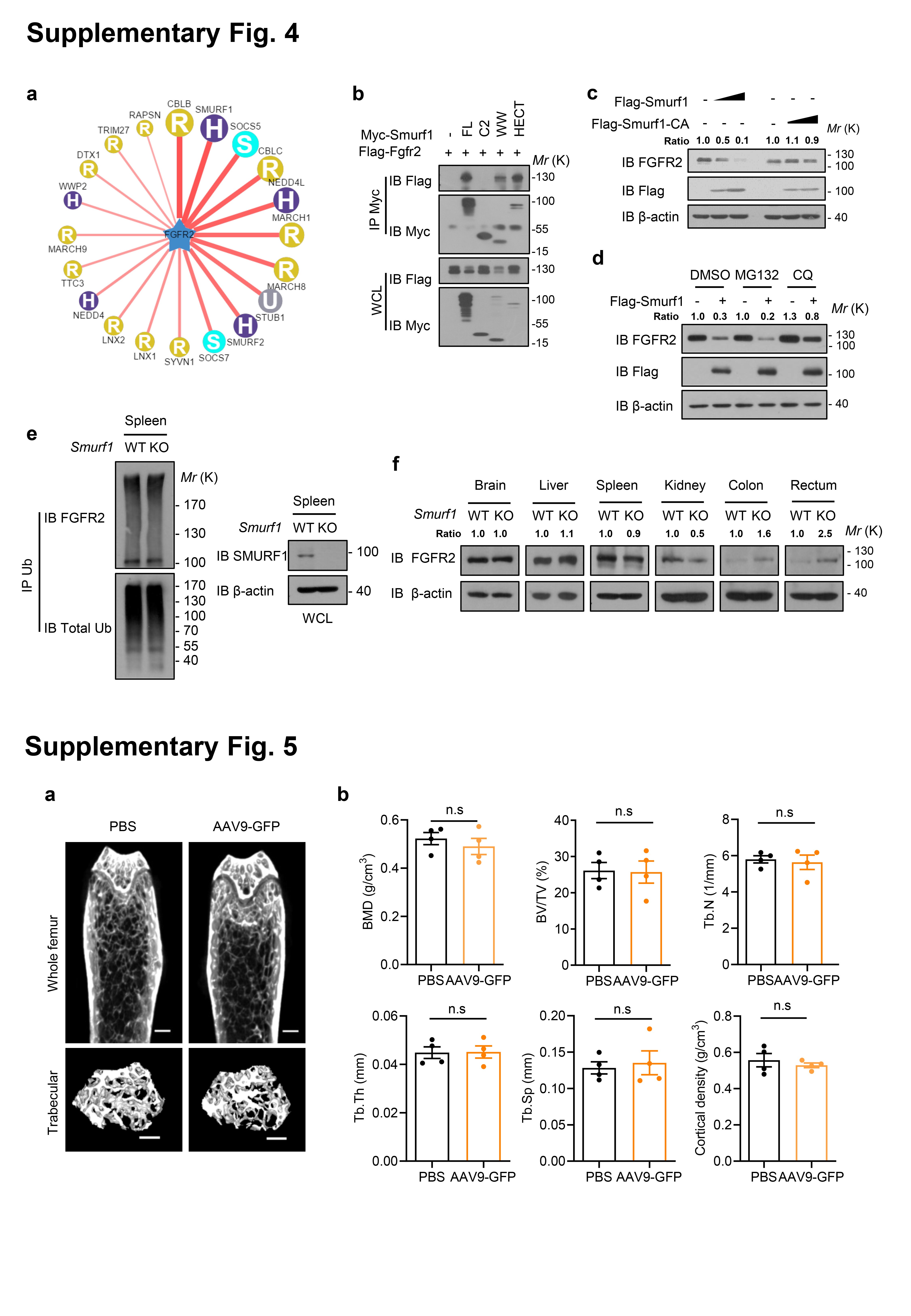


**Figure. S5 AAV9-delivered GFP did not affect the bone phenotypes of wild-type mice.**

**a** Representative micro-CT images of whole femoral (top) and trabecular (bottom) bones from wile-type mice after 2 months of AAV9-GFP injection. n = 4 per group. Scale bars, 0.5 mm.

**b** Histomorphometric analysis of trabecular bones in (**a**), including BMD, BV/TV, Tb.Th, Tb.N, Tb. Sp and cortical density. n = 4 per group.

n.s., not significant. All data are shown as the mean ± SEM.


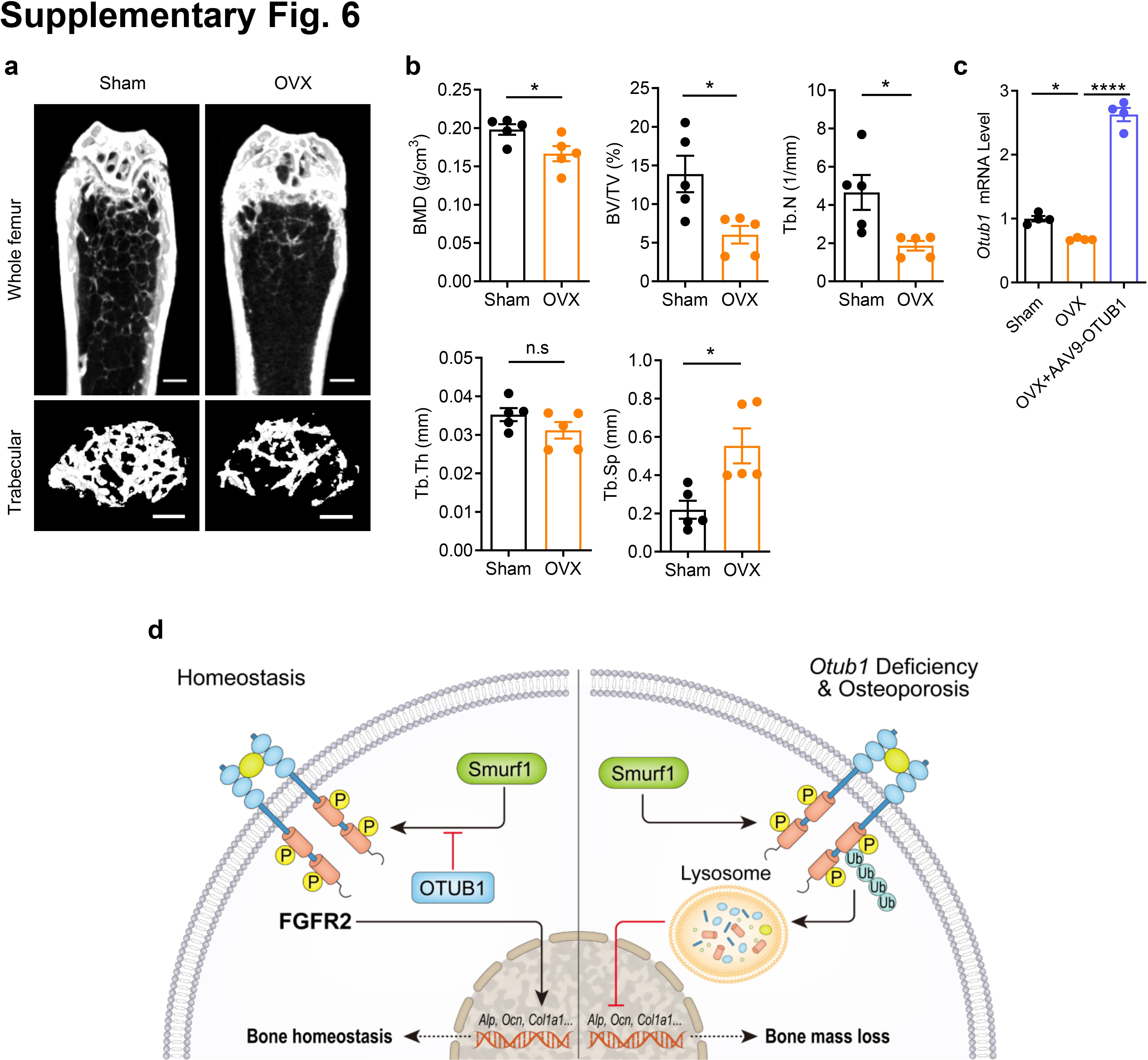


**Figure. S6. Ovariectomized mice display osteopenia.**

**a** Representative micro-CT images of whole femoral (top) and trabecular (bottom) bones from Sham and OVX mice. n = 5 per group. Scale bars, 0.5 mm.

**b** Histomorphometric analysis of trabecular bones in (**a**), including BMD, BV/TV, Tb.Th, Tb.N and Tb. Sp. n = 5 per group.

**c** Quantitative RT-PCR analysis of *Otub1* mRNA levels in femoral bones from Sham, OVX and OVX-AAV9-OTUB1 group. n = 4 per group.

**d** Schematic model of OTUB1 in promoting osteoblastic bone formation through stabilizing FGFR2. **p* < 0.05, *****p* < 0.0001, n.s., not significant. All data are shown as the mean ± SEM.

**Supplementary Table 1: Mouse qPCR primers**

| **Name** | **5' primer** | **3' primer** |
| --- | --- | --- |
| *Gapdh* | TGCACCACCAACTGCTTAG | GGATGCAGGGATGATGTTC |
| *Otub1* | CTGATGGCAACTGCTTCTACC | GGTCCTCTTTACTCTTGGCAGA |
| *Ocn* | CTCACAGATGCCAAGCCCA | CAAGGTAGCGCCGGAGTCT |
| *Osx* | GCAAGGCTTCGCATCTGAAA | AACTTCTTCTCCCGGGTGTGA |
| *Alp* | ATCTTTGGTCTGGCTCCCATG | TTTCCCGTTCACCGTCCAC |
| *Col1a1* | AGACATGTTCAGCTTTGTGGAC | GCAGCTGACTTCAGGGATG |
| *Fgfr2b* | GCAAGGTTTACAGCGATGCC | CCAGCATCCATCTCCGTCAC |
| *Fgfr2c* | CAGTGGATCAAGCACGTGGA | CGCCAAGCACGTATATTCCC |
| *Cyclin D* | GCGTACCCTGACACCAATCTC | CTCCTCTTCGCACTTCTGCTC |
| *Cdkn1a* | CCTGGTGATGTCCGACCTG | CCATGAGCGCATCGCAATC |
| *c-myc* | GCTGGACACGCTGACGAAA | TCTAGGCGAAGCAGCTCTATTT |
| *Ctsk* | GGCCAGTGTGGTTCCTGTT | CAGTGGTCATATAGCCGCCTC |
