# Supplementary figures and images for: OTUB1 promotes osteoblastic bone formation through stabilizing FGFR2
Source: Signal Transduct Target Ther. 2023 Apr 7;8:142. doi: 10.1038/s41392-023-01354-2 (PMC10079838; doi:10.1038/s41392-023-01354-2)

Source data Fig. 3

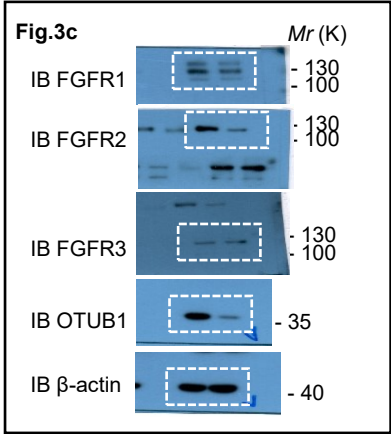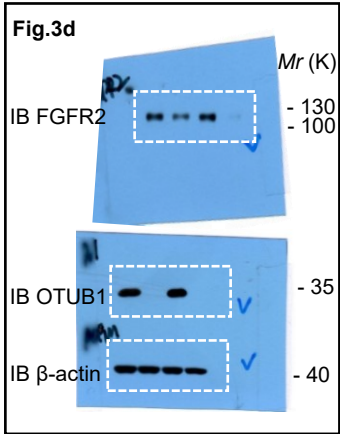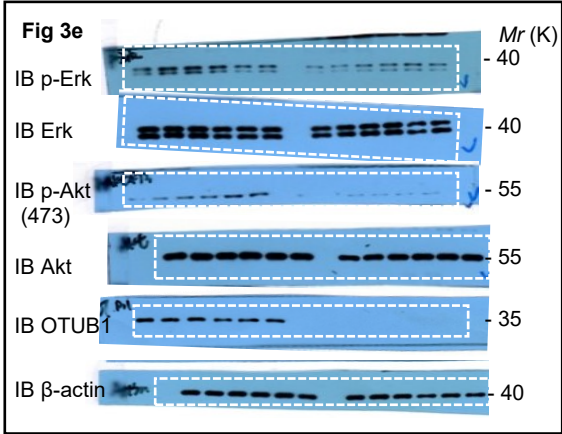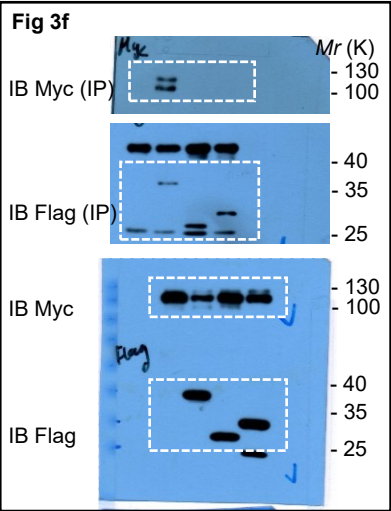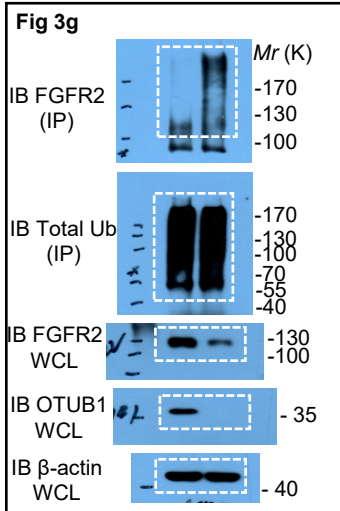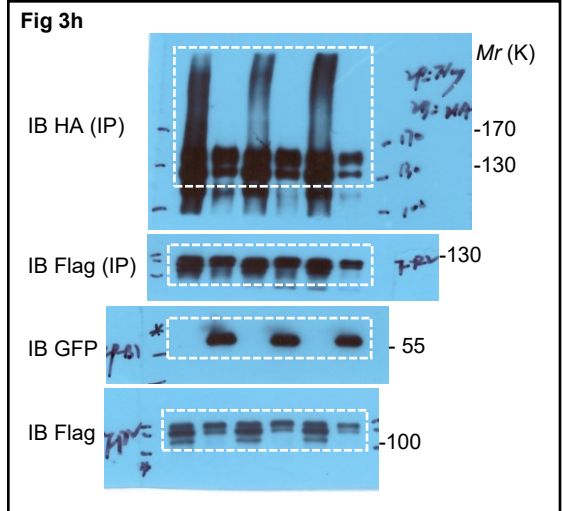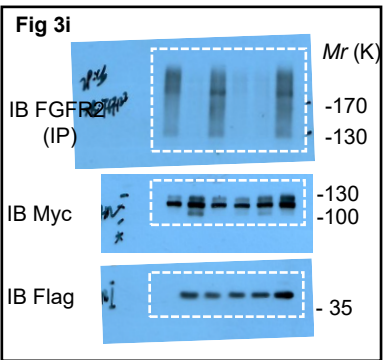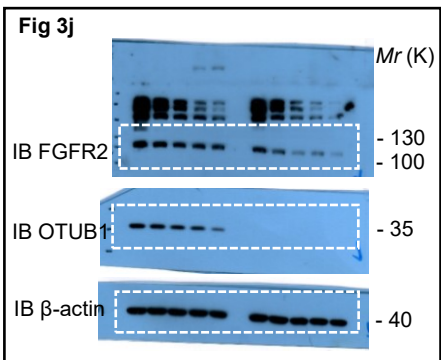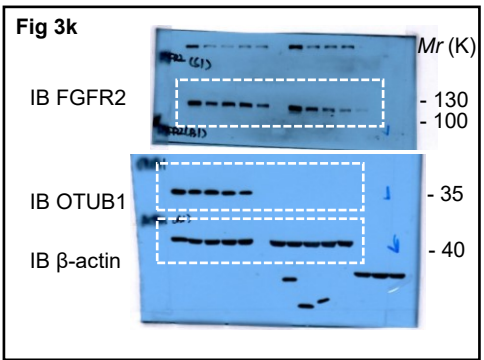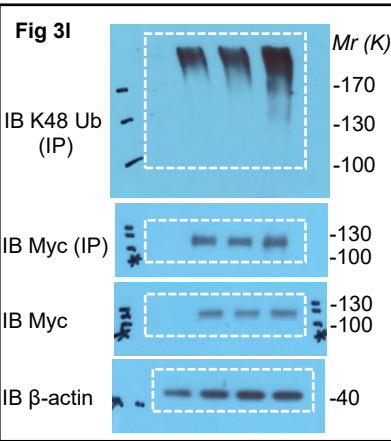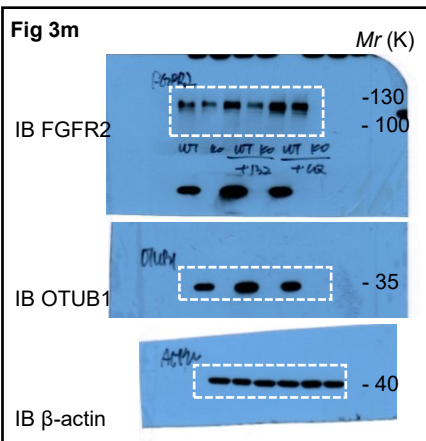

Source data Fig. 4

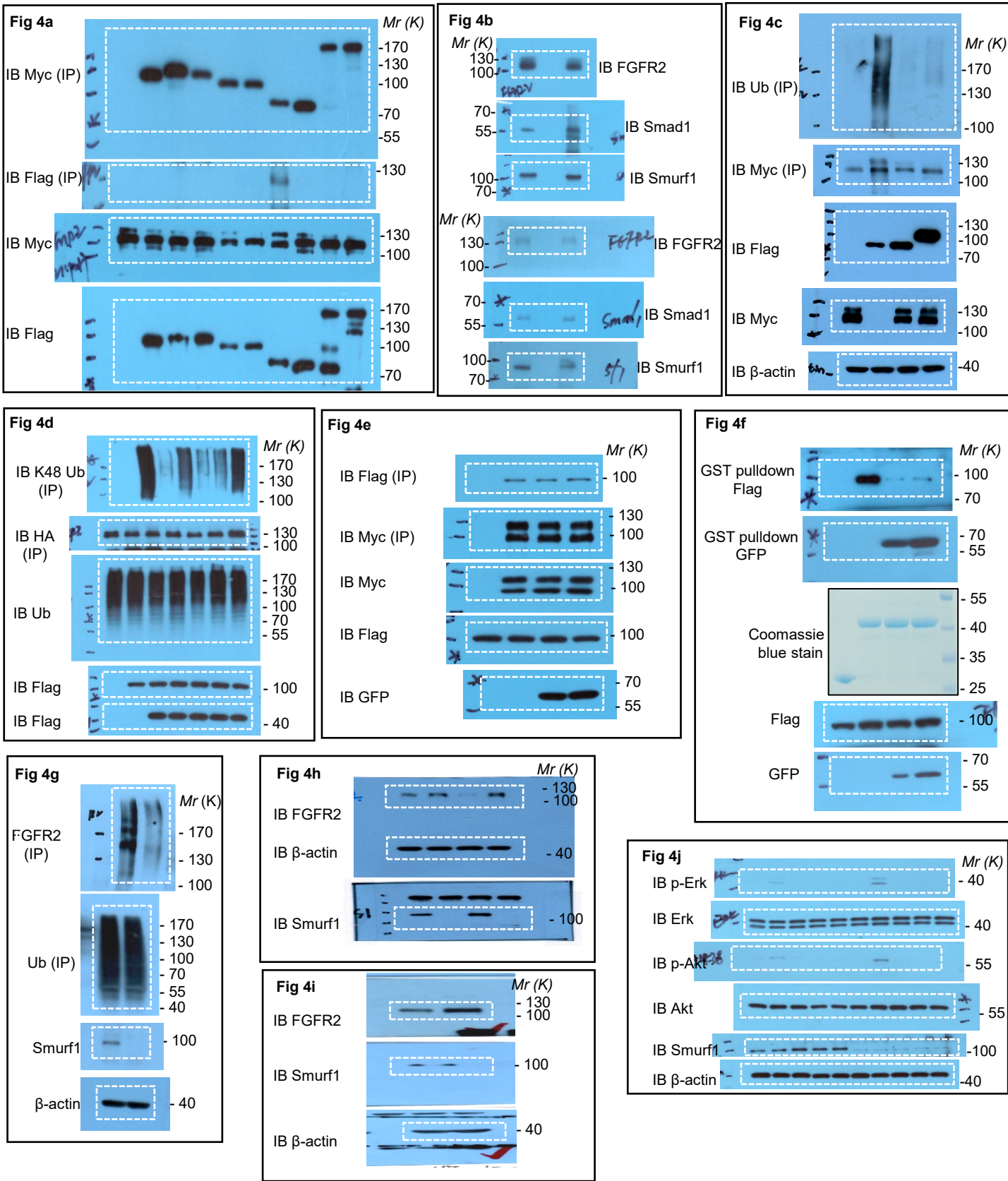

Source data Fig. S1 - S5

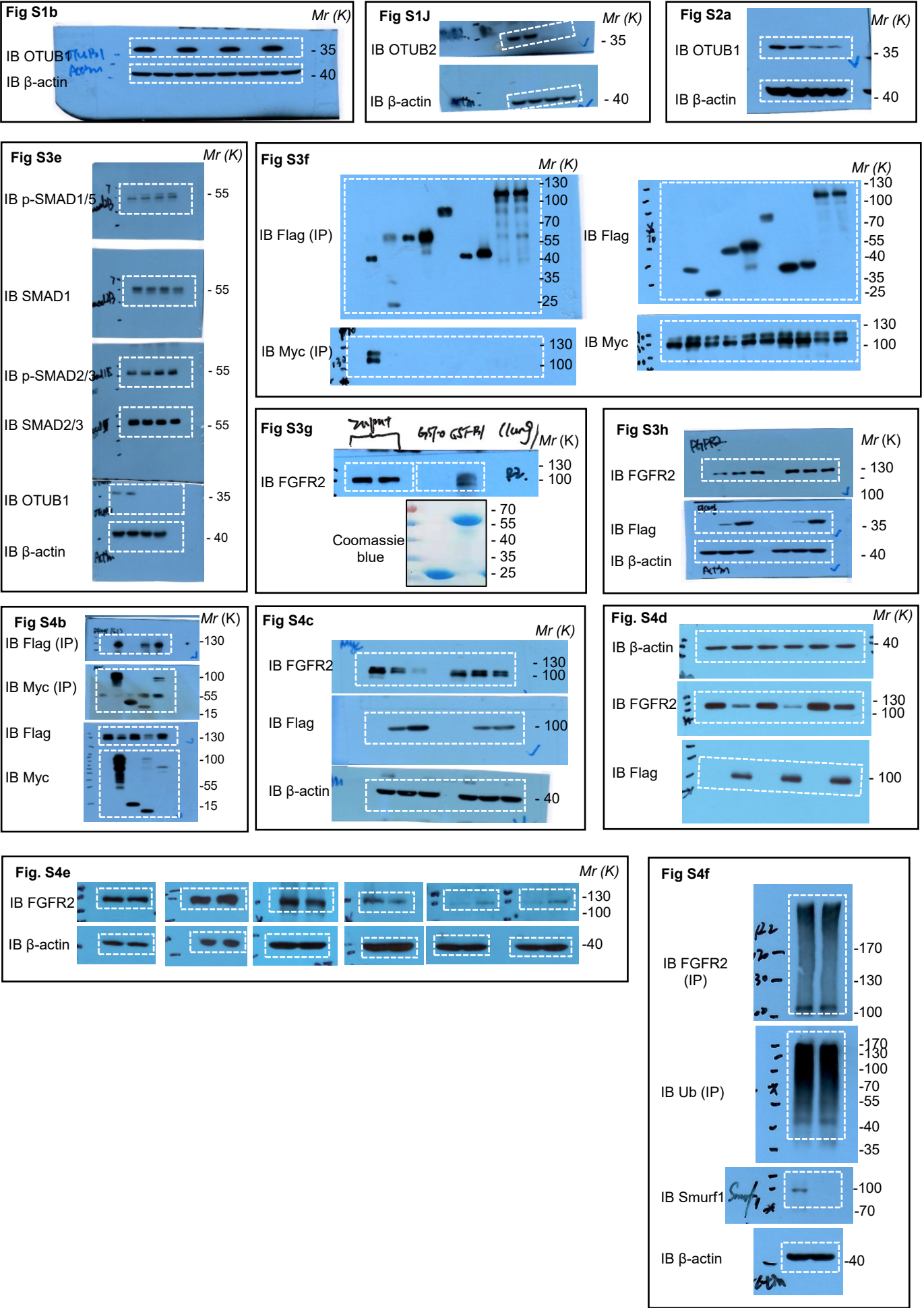

Supplement: Supplementary file 2 — Unprocessed western blot data [file 41392_2023_1354_MOESM2_ESM.pdf]
